# Rotation of sex combs in *Drosophila melanogaster* requires precise and coordinated spatio-temporal dynamics from forces generated by epithelial cells

**DOI:** 10.1371/journal.pcbi.1006455

**Published:** 2018-10-10

**Authors:** Ernest C. Y. Ho, Juan Nicolas Malagón, Abha Ahuja, Rama Singh, Ellen Larsen

**Affiliations:** 1 Department of Cell and Systems Biology, University of Toronto, Toronto, Ontario, Canada; 2 Boyer Center for Molecular Medicine, Yale University, New Haven, Connecticut, United States of America; 3 Department of Biology, McMaster University, Hamilton, Ontario, Canada; 4 College of Natural Sciences, Minerva Schools at KGI, San Francisco, California, United States of America; Mathematical Institute and the Institue for Biology, Leiden, NETHERLANDS

## Abstract

The morphogenesis of sex combs (SCs), a male trait in many species of fruit flies, is an excellent system in which to study the cell biology, genetics and evolution of a trait. In *Drosophila melanogaster*, where the incipient SC rotates from horizontal to a vertical position, three signal comb properties have been documented: length, final angle and shape (linearity). During SC rotation, in which many cellular processes are occurring both spatially and temporally, it is difficult to distinguish which processes are crucial for which attributes of the comb. We have used a novel approach combining simulations and experiments to uncover the spatio-temporal dynamics underlying SC rotation. Our results indicate that 1) the final SC shape is primarily controlled by the inhomogeneity of initial cell size in cells close to the immature comb, 2) the final angle is primarily controlled by later cell expansion and 3) a temporal sequence of cell expansion mitigates the malformations generally associated with longer rotated SCs. Overall, our work has linked together the morphological diversity of SCs and the cellular dynamics behind such diversity, thus providing important insights on how evolution may affect SC development via the behaviours of surrounding epithelial cells.

## Author summary

The sex comb (SC) is a series of modified bristles on the male forelegs of many species of fruit flies. The size, position and shape of these sex combs vary drastically across different fly species. Therefore, SCs are a model system which illustrates the interaction between evolution and organism development influencing phenotypic features. In this work, we use a combined simulation-experimental approach to study the cellular processes involved in the rotation of developing SCs in common fruit flies (*D. melanogaster*). Our results indicate that despite the appearance of a complicated set of motions of surrounding cells associated with SC rotation, the final SC attributes only depend on a few selected parameters. We showed that changes in the timing and extent of cell size increase in distal cells altered the extent of SC rotation and breakage. Furthermore, these changes were sufficient to account for the observed variations in SC rotation between different fly species. Thus, our computational model has given us important insights on how evolution may use various cellular processes as a means to manifest the diversity of SCs across different fly species.

## Introduction

Morphogenesis concerns the development of forms in organisms. It is a major theme in biology not only because of its importance in its own right, but also because of its essential relationship and interactions with biological evolution. Darwin pointed out that the adult form of an organism depends on its developmental trajectory. Hence, if heredity is important in determining the adult form, it must exert its influence during development [1]. As with many natural phenomena, physical processes involving morphogenesis inherently span many hierarchical levels of biological organization and time scales, in that events from one level (e.g. genetic level) can alter events at another level (e.g. cellular level). A central research topic in evo-devo is to understand how those hierarchical levels integrate leading to evolution [2–4].

**Fig 1:**
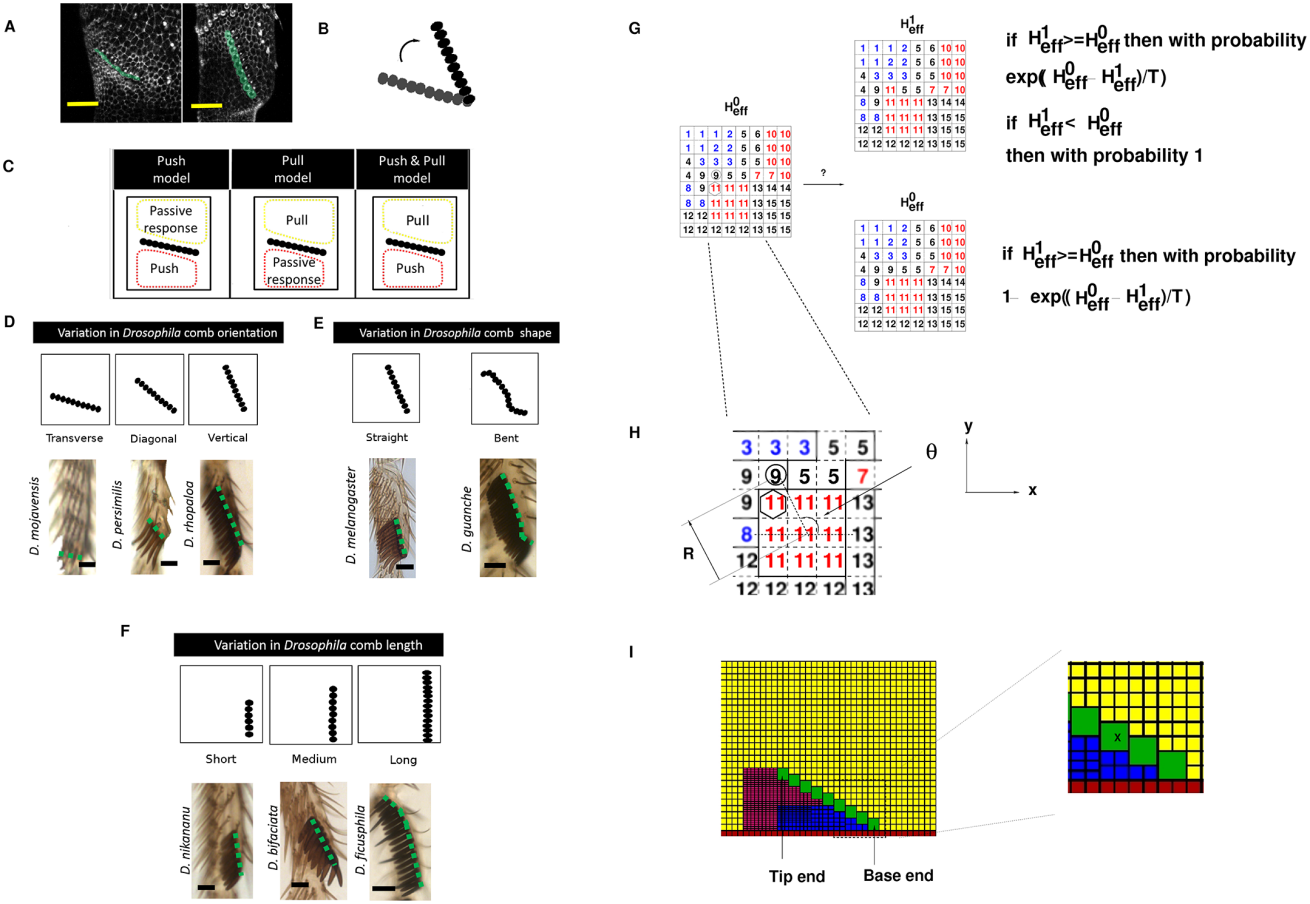
Schematics showing possible variations of SC features and illustration of the Cellular Potts Model for simulation. **A** Confocal images of ♂ wt (male wildtype) SC (labelled green) at 23 and 36 hours after pupariation. Each scale bar: 20 *μ*m. **B** Schematic showing the rotation of SC. **C** Schematics showing three hypotheses for SC rotation: the push model (left) where SC rotates due to the force generated by the expanded distal cells, the pull model (centre) where SC rotates due to the pulling force generated by the contraction of proximal cells, and the push and pull model (right) where SC rotates due to both the pushing and pulling forces from the cells distally and proximally. **D** Schematics showing possible variations in SC orientations during evolution (top). Images of adult legs of *Drosophila* species that exemplify these variations (bottom). Each scale bar: 20 *μ*m. **E** Schematics showing some possible variations in SC shapes during evolution (top). Images of adult legs of *Drosophila* species that exemplify these variations (bottom). Each scale bar: 20 *μ*m. **F** Schematics showing possible variations in SC lengths during evolution (top). Images of adult legs of *Drosophila* species that exemplify these variations (bottom). Each scale bar: 20 *μ*m. **G** Left: an example configuration of pixels in the Cellular Potts Model. Each square enclosed by dotted lines is a pixel (8 × 8 pixels in this configuration). The number inside the pixel represents the cell index label *σ*. Each pixel at a single time can only be labelled by one cell index. In this example there are 15 “cells” occupying 64 pixels at the current moment, and the solid lines represent “cell” boundaries. The colours of the cell index labels represent cell types *c*. Right: illustration of an attempted pixel label flip during a Monte Carlo step (mcs). The circled pixel on the left panel is the randomly chosen “target pixel”, and the pixel with a hexagon is the (also randomly chosen) neighbouring pixel (invading pixel). Whether there is a change to the cell index label of the target pixel is dependent on the relative effective energies of the configuration with and without the flipping. During a single mcs, there can be many such attempted pixel label flips (as specified by the parameters of the simulation–see Table 1). **H** Illustration of how variables *θ* and *R* are calculated for axial preference of epithelial cells. In this example, “cell” 11 is the “invading” cell (since the “invading” pixel belongs to that cell), and the “target” pixel is in cell 9. *θ*(*σ* = 11) is the angle subtended between the two vectors: the *x* axis and the vector $\vec{R}\left(\sigma =11\right)$ that points from the centre of mass (CoM) of the “cell” 11 to the target pixel. *R*(*σ* = 11) is the norm of $\vec{R}\left(\sigma =11\right)$. In this example only *θ*(*σ* = 11) and *R*(*σ* = 11) are shown. Similarly, *θ*(*σ* = 9) (not labelled in this figure) is the angle subtended between the *x* axis and the vector $\vec{R}\left(\sigma =9\right)$ that points from the CoM of cell 9 to the target pixel, while *R*(*σ* = 9) (again not labelled in this figure) is the norm of $\vec{R}\left(\sigma =9\right)$. **I** Left: an example initial cell configuration for a 9-tooth SC simulation. As in **G** and **H**, we use different colours to differentiate cell types (blue-EP1; magenta-EP2; yellow-EP3; green-SCT; red-BA), but the boundaries (black horizontal and vertical lines) depicted here are cell boundaries, not pixel boundaries. Right: blow-up of a selected rectangular area from the upper panel to illustrate cell types and sizes. In this magnification, there are four types of cells shown: EP1 (blue), EP3 (yellow), SCT (green) and BA (red). Cells of the same type may have different initial areas, as demonstrated by the blue EP1 cells. As an indication of the relative initial areas occupied by different cells, the (square) SC tooth marked with an “X” has an area of 6 × 6 = 36 pixels. “Proximal” refers to the region above the SC and “distal” refers to the region below the SC. Therefore, EP1 and EP2 cells are sometimes called “distal cells” but EP3 cells are sometimes called “proximal cells”.

One example of morphogenesis that lends itself to exploring physical processes at genetic, cellular and tissue levels through time is the formation of a morphological feature called the sex comb (SC) in species of the *Drosophilidae* family (Fig 1A) [5–7]. Existence of SCs is a male-specific trait of many species of flies. The phylogenetic relationships of these species have been studied showing that SCs have evolved independently several times [8–10]. In *D. melanogaster*, the SC is a linear, almost vertical arrangement of modified bristles on the first-tarsal segment of their forelegs. During development, SCs of *D. melanogaster* were imputed to rotate from a horizontal to an almost vertical position (Fig 1B) [11] and this has been corroborated using modern genetic and microscopical tools [6, 12–14]. In related experiments, Malagón [15] had initial evidence that the major force driving SC rotation was provided by cell expansion distal to (below) the SC, and that the cells proximal to (above) the SC passively responded by diminishing in area and disappearing from the epithelium.

**Table 1:** System-wide simulation parameters.

Parameter	Units	Values
“Temperature” parameter *T*	-	100
Neighbour order	-	2
Number of attempted pixel flips per mcs	-	9576 (38304 for Figure I in S1 Text)
Length of each simulation	mcs	2000

However, not all species of flies with vertical, linear SCs develop them through rotation. Some species utilize different bristles which are already in a vertical position and merely have to come together to form a vertical column. Indeed some species with vertical SCs on more than one segment use a different method on each segment [8]. *Drosophila* SCs display spectacular developmental and morphological variations during evolution. Some examples include comb shape (Fig 1E), comb length (Fig 1F), number of combs per tarsal segment, tooth size and pigmentation. Possibly, the most interesting comb feature involves its orientation [9], which constantly changes between three positions relative to joint: transverse, diagonal, and vertical (Fig 1D). Malagon and Larsen [16] suggest that genetic perturbations in *D. melanogaster* can easily phenocopy changes in comb variation. Thus, the SC system provides a rich developmental and evolutionary phenomenology with which to explore the strategies and tactics involved in morphogenesis and its evolution. Understanding the dynamics of cell behaviours and the mechanical constraints underlying SC morphogenesis represents an important step towards linking the genetics of cellular behaviours which occur during development to their evolution over time.

Combined use of different approaches is essential for further progress in evolutionary-developmental biology. We previously used a combination of developmental and experimental approaches and showed the role of developmental constraints and interaction between development and selection in the rotation and evolution of SCs in *D. melanogaster* [6]. Here, we use a combination of computational modelling (cellular Potts model, or CPM, [17]) with experimental evidence to investigate and quantify the spatio-temporal dynamics and interplay of various mechanical characteristics of cells critical for the proper rotation of SCs in *D. melanogaster*. Although computational modelling or a hybrid computational-experimental approach has been used successfully to describe various cellular processes, including examples in morphogenesis [18–26], to our best knowledge this work represents the first hybrid approach used to explore the cellular dynamics driving SC morphogenesis. Furthermore, we emphasize that in addition to replicating experimental results in our simulations, we have generated quantitative and falsifiable hypotheses which can guide future experiments, thus showing the synergy between experiments and computational modelling in generating novel insights in broad areas of biology.

## Results

As the SC rotates during development, pronounced changes in apical cell area take place between the initial and final stages of sex comb rotation, while during these same stages epithelial cell division is conspicuously absent [15, 16, 27, 28]. Based on the temporal variation in cell parameters in which epithelial cells from the distal region increase in size but cells from the proximal region decrease in size, at least three hypothetical models can be envisioned: pull, push, and push and pull [15] (Fig 1C). In the push model, the cells in the region distal to the sex comb actively increase in apical cell area, thus pushing the sex comb, and in turn rearranging the proximal region as a passive response. In contrast, in the pull model, the hypothesized active contraction of proximal cells generates a pulling force on the SC during rotation. Malagón [15] described the cellular dynamics taking place in both proximal and distal regions to the combs and found different lines of evidence in favor of the push model. At the same time, the lack of observation of a clear temporal pattern of extrusion of proximal cells from the epithelial tissue, and the apparent absence of a spatial pattern of proximal cell area distribution mean that the pull model is experimentally less likely to have taken place [15, 27, 29]. (Please see “Discussion” for an elaboration of experimental observations against the pull model.) To test and expand the push model of rotation, we perform experiments and simulations to study SC rotation in male *D. melanogaster*. We hypothesize that SC rotation is the result of the directional dependent expansion of distal epithelial cells [15], with the distal epithelial cells elongating along the y-axis. Therefore, our aim is to uncover the spatio-temporal dynamics of the epithelial cells underlying the rotation of SCs. (Please see Fig 1G, 1H and 1I and “Materials and methods” for details of our methods and models and also the definitions of terms. In particular, Fig 1I has the colour scheme of the different cell types used for our simulations, and Eqs 5 and 7 that describe the expansion of epithelial cells.)

All our simulations described in this work are based on the formalism of cellular Potts Model in which an effective Hamiltonian (Eq 1) describes the energy state of the system to be simulated (i.e. the SC and its surrounding cells). The distal cells (EP1 and EP2 cells) are small at start and the SC is positioned almost horizontally (Fig 1I). We then make the distal cells increase their target area (Eq 5) over the course of the simulation, causing them to swell and push the SC (the push model). The distal cells also elongate in the direction of the y-axis (Eq 7), consistent with the experimental observation that distal cells are polarized during SC rotation. As well, proximal cells (EP3 cells) may passively extrude from the tissue (see “Materials and methods”) as a consequence of being pushed by other cells, in accordance with experimental observations.

Our presentation is structured as follows. First, we use the simulated rotations of 9-tooth SCs to show that proper rotation is critically dependent on the initial spatial arrangement of distal cells. In these simulations we determined that the expanding distal cells have to be arranged in a manner that delivers an inhomogeneous and differential push in the direction perpendicular to the length of the SC, with increasing pushing force farther away from the base (pivot point of rotation) of the SC. Specifically, distal cells closer to the tip of the SC start out with much smaller apical areas than distal cells closer to the base, but both eventually expand to comparable sizes. We then use these optimal initial epithelial cell configurations as the starting point to investigate the effects of SC length and adhesion between SC teeth on proper SC rotations. We discover that variations in length and adhesion of SCs have significant effects on both the orientation, curvature and the overall success of SC rotation. Importantly, there are increased instances of SC breaking during rotation as it is getting longer, even with the optimal initial spatial epithelial cell configuration conducive for proper rotation. Finally, we demonstrate how the breaking of long SCs can be partially rescued by invoking a temporal sequence of epithelial cell expansion, in addition to the already discussed spatial arrangement of epithelial cells.

### Proper rotation of sex comb is dependent on a tight initial spatial arrangement of distal epithelial cells

**Fig 2:**
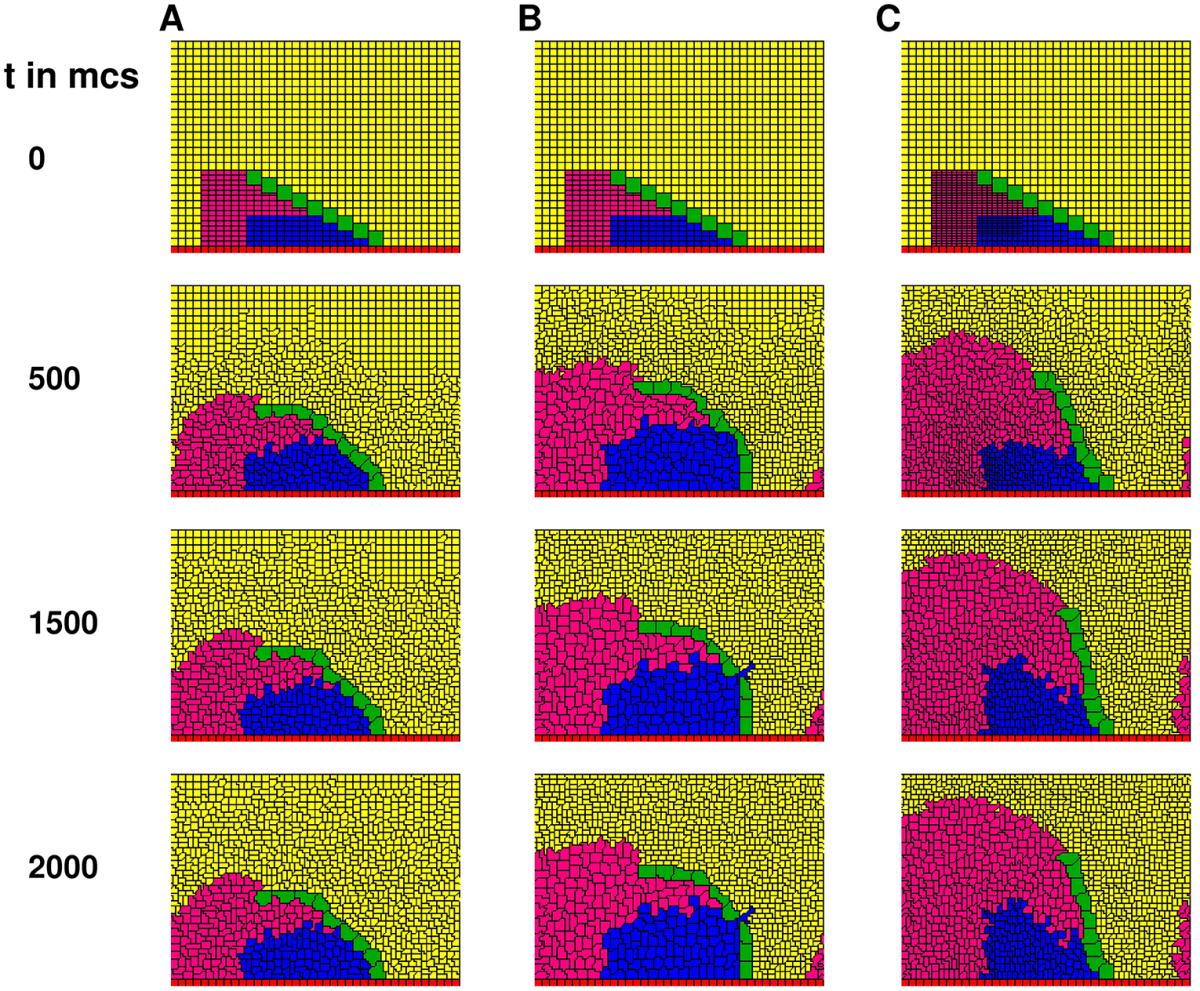
Inhomogeneous and differential epithelial cell expansion critical for proper SC rotation. **A,B** Approximately homogeneous spatial arrangement of distal epithelial cells. Adhesion parameter *J*(*SCT*, *SCT*) used: 4000. **C** Inhomogeneous spatial arrangement of distal epithelial cells. Adhesion parameter *J*(*SCT*, *SCT*) used: 4000. Please see S1 Video for a frame-by-frame capture of this simulation.

In this section we show via simulation how the initial spatial arrangement of distal cells is critical for proper SC rotation. Fig 2A and 2B show snapshots of two SC rotation simulations. These two examples share identical initial spatial cell configurations. Importantly, under this specific initial cell configuration, every distal cell has an initial cell size roughly equal to each other (when *t* = 0 mcs, top panels of Fig 2A and 2B). Moreover, $a_{target}^{term}$ (Eq 5) is set to be equal for every distal cell in each simulation of Fig 2A and 2B. The only difference in parameter setup between Fig 2A and 2B is that $a_{target}^{term}$ of distal cells of Fig 2A is smaller than that of Fig 2B. ($a_{target}^{term}\left(c=EP1\right)=a_{target}^{term}\left(c=EP2\right)=13$ pixels in Fig 2A, while $a_{target}^{term}\left(c=EP1\right)=a_{target}^{term}\left(c=EP2\right)=20$ pixels in Fig 2B.) Taken together, expansion rates of distal cells are different across simulations (and with Fig 2B having a higher expansion rate than Fig 2A), even though the expansion rates are roughly uniform across distal cells within a simulation.

It is clear that proper rotation is unlikely to occur with such uniform expansion rates of distal cells, as evidenced by the severe curvature of (Fig 2A) or broken (Fig 2B) SC at the end of rotation. These rotation defects are not rescued by a blanket change in expansion rates of distal epithelial cells (SC rotations in both Fig 2A and 2B are defective). The curvatures of the rotated SCs suggest that uniformity of expansion rate across epithelial cells causes unequal rotation along the length of the SC, with the base end receiving more rotation than the tip end.

To restore proper rotation, distal cells have to be arranged in a manner that provides more rotation to the SC tip. One way to achieve this is to make the initial sizes inhomogeneous across distal cells, with the distal epithelial cells having smaller initial sizes closer to the tip end of the SC (top panel of Fig 2C). When coupled with similar $a_{target}^{term}$ values (Table 2) across distal cells, this inhomogeneous spatial arrangement of epithelial cells creates a differential push which largely maintains the shape of the SC during the entire rotation, therefore increasing the likelihood of proper SC rotation (Fig 2C).

**Table 2:** Mechanical parameters of different cell types for simulations, unless otherwise specified in the main text. A value of “A” for *a*_*target*_(*c*, *t* = 0) indicates that the initial area target value is the same as the initial area of that cell.

Parameter	Units	Values			
		*c* = EP1	*c* = EP2	*c* = EP3	*c* = SCT
*a*_*target*_(*c*(*σ*), *t* = 0)	pixel	A		9	36
$a_{target}^{term}\left(c\right)$	pixel	9 (5-tooth)	13 (5-tooth)	5	36
		9 (7-tooth)	13.2 (7-tooth)		
		9.8 (9-tooth)	12.8 (9-tooth)		
		9.8 (11-tooth)	12.6 (11-tooth)		
λ_*a*_(*c*)	pixel^−2^	1 × 10^4^		500	1 × 10^4^
*τ*(*c*)	mcs	100 (5-tooth)			N/A
		160 (7-tooth)			
		220 (9-tooth)			
		280 (11-tooth)			
*ϵ*(*c*)	-	0.03		0	
*l*_*target*_(*c*, *t* = 0)	${\text{pixel}}^{\frac{1}{2}}$	$\left\lceil \sqrt{a_{target}\left(c,t=0\right)}\right\rceil$			
λ_*l*_(*c*)	pixel^−1^	3		0	1000
*J*	-	see Table 3			

Even though we have shown only three examples, the above phenomenon is robust in all simulations. In fact, we are unable to produce a normally straight and vertical SC in any simulation with initial spatial cell configurations and $a_{target}^{term}$ values that represent uniform expansion rates across distal epithelial cells. Proper SC rotations can only occur in simulations with inhomogeneous distal epithelial cell expansion rates in the manner as in Fig 2C. We thus deduce that such inhomogeneous expansion rates amongst distal epithelial cells, brought about by the inhomogeneous initial spatial cell configuration, must be a critical biological mechanism underlying proper SC rotation.

### Dependence of rotated SC orientation on expansion characteristics of epithelial cells

**Fig 3:**
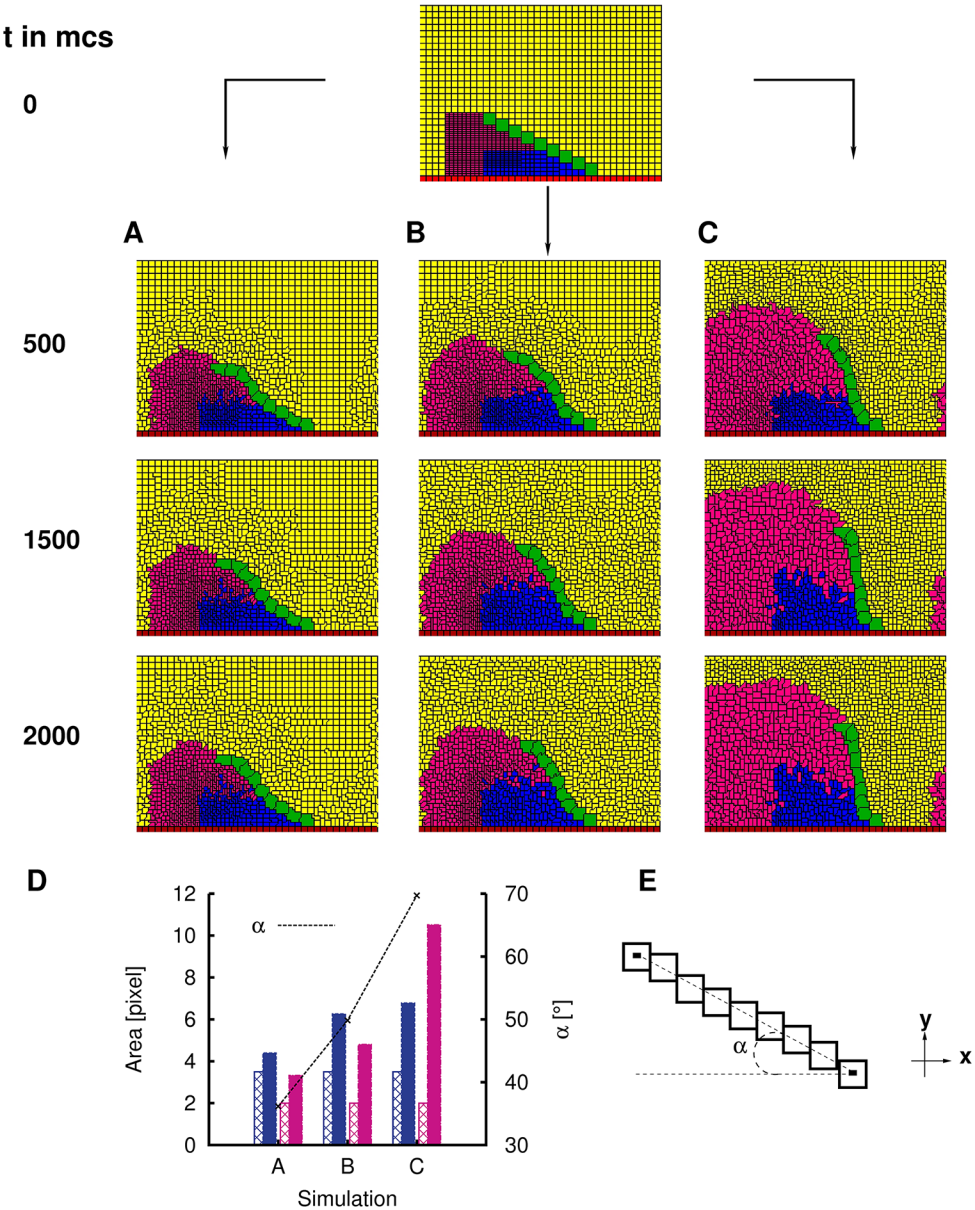
SC rotation angle is dependent on expansion characteristics of epithelial cells. **A** Representative simulation of a minimally rotated SC upon completion of rotation. Adhesion parameter *J*(*SCT*, *SCT*) used: 0. **B** Representative simulation of an intermediately rotated SC upon completion of rotation. Adhesion parameter *J*(*SCT*, *SCT*) used: 0. **C** Representative simulation of a maximally rotated SC upon completion of rotation. Adhesion parameter *J*(*SCT*, *SCT*) used: 0. **D** Summary statistics of the change in areas of distal epithelial cells in each of simulations A, B and C. Vertical bars represent mean areas of distal epithelial cells. Colours correspond to the type of cells depicted in simulations. Non-solid bars represent values calculated at the start of simulations and solid bars represent values calculated at the conclusion of simulation (*t* = 2000 mcs). Dotted line represents *α*, final SC angle of each of the simulations. **E** Graphical illustration of calculation of rotation angle *α*. A straight line is connected between the CoM of the SC segments that are located at the two extreme ends of the SC. *α* is the angle between this straight line and the -x-axis.

Having established that the initial spatial configuration of epithelial cells is crucial for proper SC rotation, we now investigate the dependence of SC orientation on the expansion parameters of epithelial cells. Fig 3A, 3B and 3C show respectively three representative SC simulations in which we obtain increasing final rotation angles *α* (definition in Fig 3E) while controlling expansion rates of distal cells. In each of the three simulations, we start with the common initial condition (top panel of Fig 3) where the starting areas of distal cells are different, with the larger cells concentrated towards the base (pivot point) of the SC and the smaller cells concentrated towards the SC “tip”. This common initial condition used here is identical to the one used in Fig 2C.

To obtain different final rotated SC angles *α*, we control the expansion characteristics by using different values of $a_{target}^{term}$ for distal cells in each of the three representative simulations. We set smaller values of $a_{target}^{term}$ to obtain a smaller *α*, and vice versa. Specifically, we set $a_{target}^{term}\left(c=EP1\right)=6.5$ and $a_{target}^{term}\left(c=EP2\right)=5$ in Fig 3A; $a_{target}^{term}\left(c=EP1\right)=9$ and $a_{target}^{term}\left(c=EP2\right)=7$ in Fig 3B. In Fig 3C, the default values of $a_{target}^{term}$ are used (Table 2). Spatially inhomogeneous distal cell expansion is observed in each of these three simulations from the summary statistics Fig 3D. Specifically, in each of these three simulations, the magenta distal cells EP2 (closer to the SC “tip”) expand the most and the blue distal cells EP1 (closer to the base pivot point) expand the least. As discussed in Fig 2C, such spatially inhomogeneous and differential expansion in distal epithelial cells is critical for providing the appropriate amount of push across the length of SC, so that it is still well-formed and intact towards the end of the rotation. We illustrate here that the degree of the inhomogeneity determines the final SC rotation angle *α*. An SC rotation with a higher angle *α* requires a higher spatial inhomogeneity of expansion between distal cells at each of the extreme ends of SC, as one readily observes from the summary statistics that the spatial inhomogeneity in distal cell expansion is highest in Fig 3C but lowest in Fig 3A.

**Fig 4:**
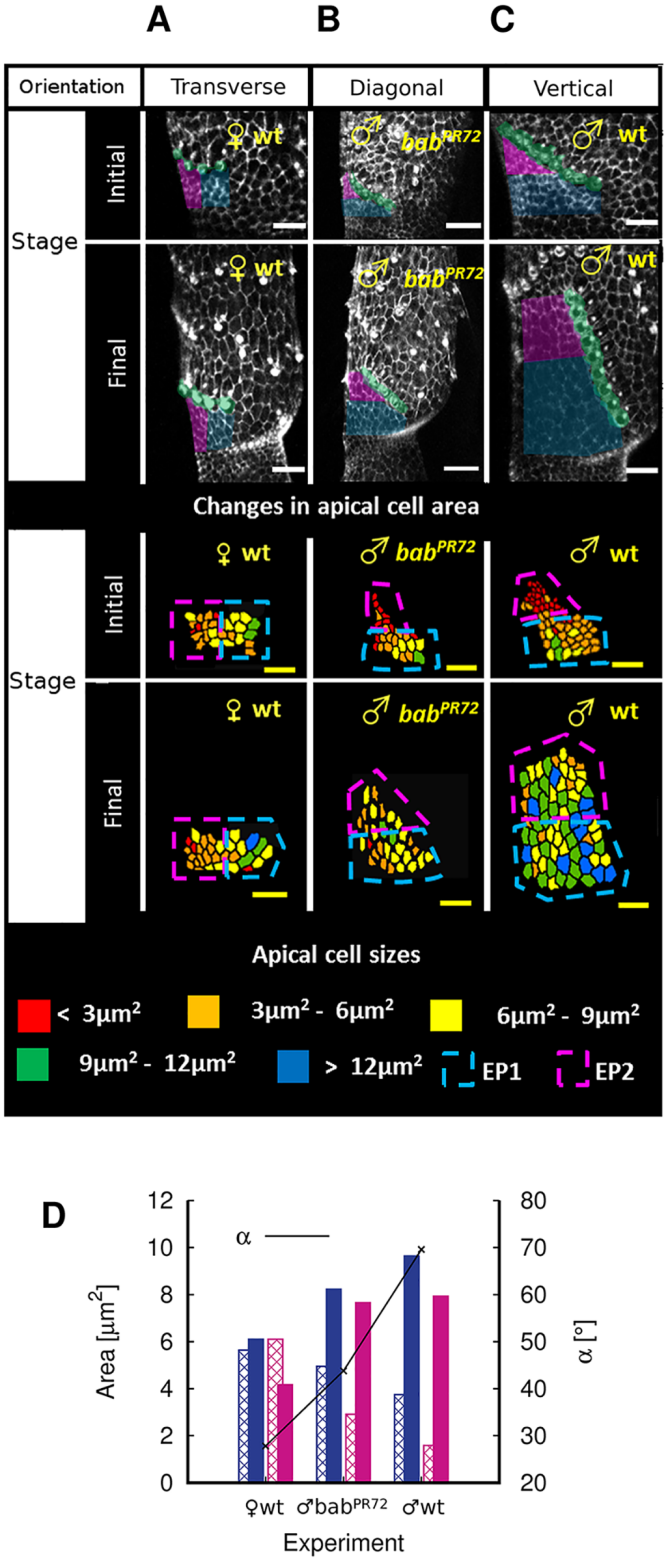
Confocal images of SC experiments for ♀wt, ♂*bab*^*PR*72^ and ♂wt. Each scale bar: 10 *μ*m. Initial = 23 hours AP; Final = 36 hours AP. Please see S2 Video. for a frame-by-frame capture of the entire SC rotation process of three flies with identical genotypes as the three here. **A** Minimally rotated SC in female wildtype. **B** Intermediately rotated SC in mutant *bab*^*PR*72^. **C** Maximally rotated SC in male wildtype. **D** Summary statistics of the change in areas of distal epithelial cell in experiments with the above three fly genotpyes: ♀wt, ♂*bab*^*PR*72^ and ♂wt. Conventions as in Fig 3D. The values displayed here are the average values of the 5 pupae for each genotype.

Do we find spatial heterogeneity during distal cell expansion as predicted from our simulations? To test this, we performed *in vivo* experiments with ♀wt, ♂*bab*^*PR*72^ and ♂wt (Fig 4). SC rotation is absent from ♀wt, while rotation is partial in ectopic heterozygous *bab*^*PR*72^ mutants with sex comb on the second tarsal segment t2. Although five pupae were measured for each of ♀wt, ♂*bab*^*PR*72^ and ♂wt for statistical analyses (Table A in S1 Text), we only display images taken at the start and at the end of the rotation of the same representative pupa for each of ♀wt, ♂*bab*^*PR*72^ and ♂wt respectively. Following our conventions, we label the distal cells closer to the SC base as “EP1” (blue), the distal cells closer to the SC tip as “EP2” (magenta). The line separating EP1 and EP2 cells is located at the half length mark of the SC on every image on which area analysis was performed. Three observations are clear from Fig 4: 1) In rotating sex combs studied (♂wt and ♂*bab*^*PR*72^), average initial apical area (obtained by dividing the coloured area with the number of cells in the area) of EP2 (magenta) is smaller than that of EP1 (blue), showing the spatial inhomogeneity of initial apical areas amongst distal cells (median $Pr(\frac{EP2_{Initial}}{EP1_{Initial}}<1)=1.00$ with $Pr(\frac{EP2_{Initial}}{EP1_{Initial}}<1)>0.5$ at least 99.98% of times for ♂wt; median $Pr(\frac{EP2_{Initial}}{EP1_{Initial}}<1)=0.99$ with $Pr(\frac{EP2_{Initial}}{EP1_{Initial}}<1)>0.5$ at least 99.98% of the times for ♂*bab*^*PR*72^), while spatial inhomogeneity of distal cell areas is not apparent in ♀wt. (Median $Pr(\frac{EP2_{Initial}}{EP1_{Initial}}<1)∼0.54$ with $Pr(\frac{EP2_{Initial}}{EP1_{Initial}}<1)>0.5$ approximately 65% of times–S1 Text, Table B in S1 Text and Figure A in S1 Text). 2) In rotating sex combs studied (♂wt and ♂*bab*^*PR*72^), EP2 on average expands at a faster rate (defined as $\frac{\text{final}\;\text{area}}{\text{initial}\;\text{area}}\times \frac{1}{\text{time}\;\text{taken}}$) than EP1, providing a differential push to the SC during rotation (median *Pr*(*C*_*inhomogeneity*_ > 1) = 1.00 with *Pr*(*C*_*inhomogeneity*_ > 1) > 0.5 at least 99.98% of the times for ♂wt, median *Pr*(*C*_*inhomogeneity*_ > 1) = 1.00 with *Pr*(*C*_*inhomogeneity*_ > 1) > 0.5 at least 99.98% of times for ♂*bab*^*PR*72^–Table B in S1 Text and Figure A in S1 Text), and 3) how much EP2 expands faster on average than EP1 is related to *α*, with a higher *α* correlated with a greater disparity in expansion rates between EP2 and EP1 (median *Pr*(Δ*C*_*inhomogeneity*_(♂*wt*, ♀*wt*) > 0) = 1.00 with *Pr*(Δ*C*_*inhomogeneity*_(♂*wt*, ♀*wt*) > 0) > 0.5 at least 99.98% of the times; median *Pr*(Δ*C*_*inhomogeneity*_(♂*wt*, ♂*bab*^*PR*72^) > 0) = 0.70 with *Pr*(Δ*C*_*inhomogeneity*_(♂*wt*, ♂*bab*^*PR*72^) > 0) > 0.5 90% of the times; median *Pr*(Δ*C*_*inhomogeneity*_(♂*bab*^*PR*72^, ♀*wt*) > 0) = 0.99 with *Pr*(Δ*C*_*inhomogeneity*_(♂*bab*^*PR*72^, ♀*wt*) > 0) > 0.5 at least 99.98% of the times–Figure A in S1 Text and Table B in S1 Text), further buttressing the differential push claim in simulations. (*Pr* means probability. Please see, for example [30–32], for Monte Carlo simulations and bootstrapping method for statistical analyses.)

### Dependence of SC rotation on comb length and adhesion between SC teeth

**Fig 5:**
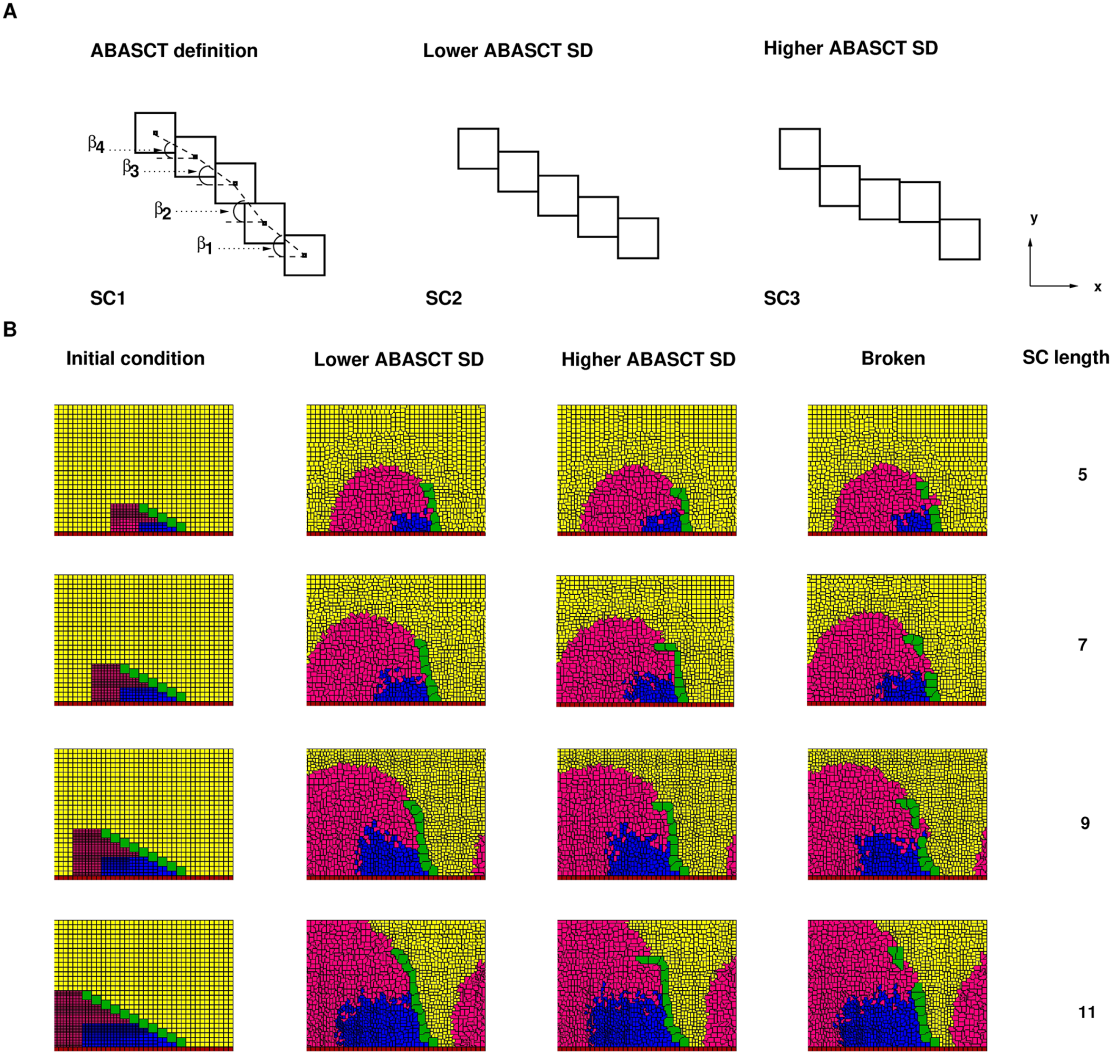
Simulation of rotation of SCs of different lengths and adhesion parameters. **A** Illustration of ABASCT (“angle between adjacent sex comb tooth”) and how it is related to the curvature of SC. **B** Initial conditions and example SC rotation simulations of different lengths. Left panel of each row: initial spatial configuration for SC rotation simulations. Second from left: rotated SC with low ABASCT SD. Third from left: rotated SC with higher ABASCT SD. Second from right: broken rotated SC. Right: Simulated SC length in number of SC teeth. Adhesion parameter *J*(*SCT*, *SCT*) used in the examples shown: 8000 for 5-tooth and 7-tooth SCs, 6000 for 9-tooth SCs, 4000 for 11-tooth SCs.

Next, we investigate the dependence of SC rotation on the length of the SC and the adhesion between successive SC teeth. To quantify SC rotation, we have already introduced *α* in the previous section and used it in Fig 3 to describe how much SCs rotate. We now introduce ABASCT, angle between adjacent sex comb teeth, which is useful to quantify the departure of rotated SCs from linearity. In Fig 5A, we illustrate the standard deviation (SD) of ABASCT is related to SC shape variation. *β*_1_, *β*_2_, *β*_3_ and *β*_4_ are respectively the angles between SC teeth 1 and 2, 2 and 3, 3 and 4, and 4 and 5 of example SC1. The SD of these angles is a measure of curvature of SCs. An almost straight SC, SC2, has a lower ABASCT SD value, but an SC that is highly non-linear, such as SC3, has a relatively large ABASCT SD value. We emphasize that ABASCT SD and *α* measure separate properties of the SC. For example, SC2 and SC3 have identical *α* but distinct ABASCT SDs.

#### Rotated SCs vary in shape and form

Fig 5B shows initial spatial cell configurations and example simulations of SC rotations with different SC lengths. Each row (from top to bottom) depicts respectively situations of 5, 7, 9 and 11-tooth SCs. The left panel of each row shows the initial spatial cell configuration corresponding to each type of simulation (5, 7, 9 and 11-tooth SC). A common feature across these four initial cell configurations is the spatial inhomogeneity in sizes of the distal cells, with the smaller distal epithelial cells concentrated closer to the SC tip, and the larger distal cells concentrated towards the SC base (pivot point). The spatial inhomogeneities in initial sizes of distal cells are calibrated such that proper and full rotation of simulated SCs is achievable (Figure B in S1 Text). Unless otherwise stated, we perform 9 sets of simulations with different adhesion parameters between SC teeth, while keeping other parameters fixed (see “Post simulation data analyses”), for each of the four SC lengths. Therefore, there are altogether 36 sets of simulations for statistical analyses of this section. The next three panels of each row depict possible scenarios of rotated SCs at the conclusion of the simulation (*t* = 2000 mcs). Each of these three simulation examples depicted in each row is drawn within the same set of 48 simulations. In other words, these simulations from the same row share the same parameters but different seeds for pseudo random number generation. The second from the left is an example in which the rotated SC has a lower ABASCT SD, the third from the left is one in which the rotated SC has a higher ABASCT SD. We also show a broken SC example in the panel second from the right.

#### SC length and adhesion on the breaking statistics of rotated SCs

**Fig 6:**
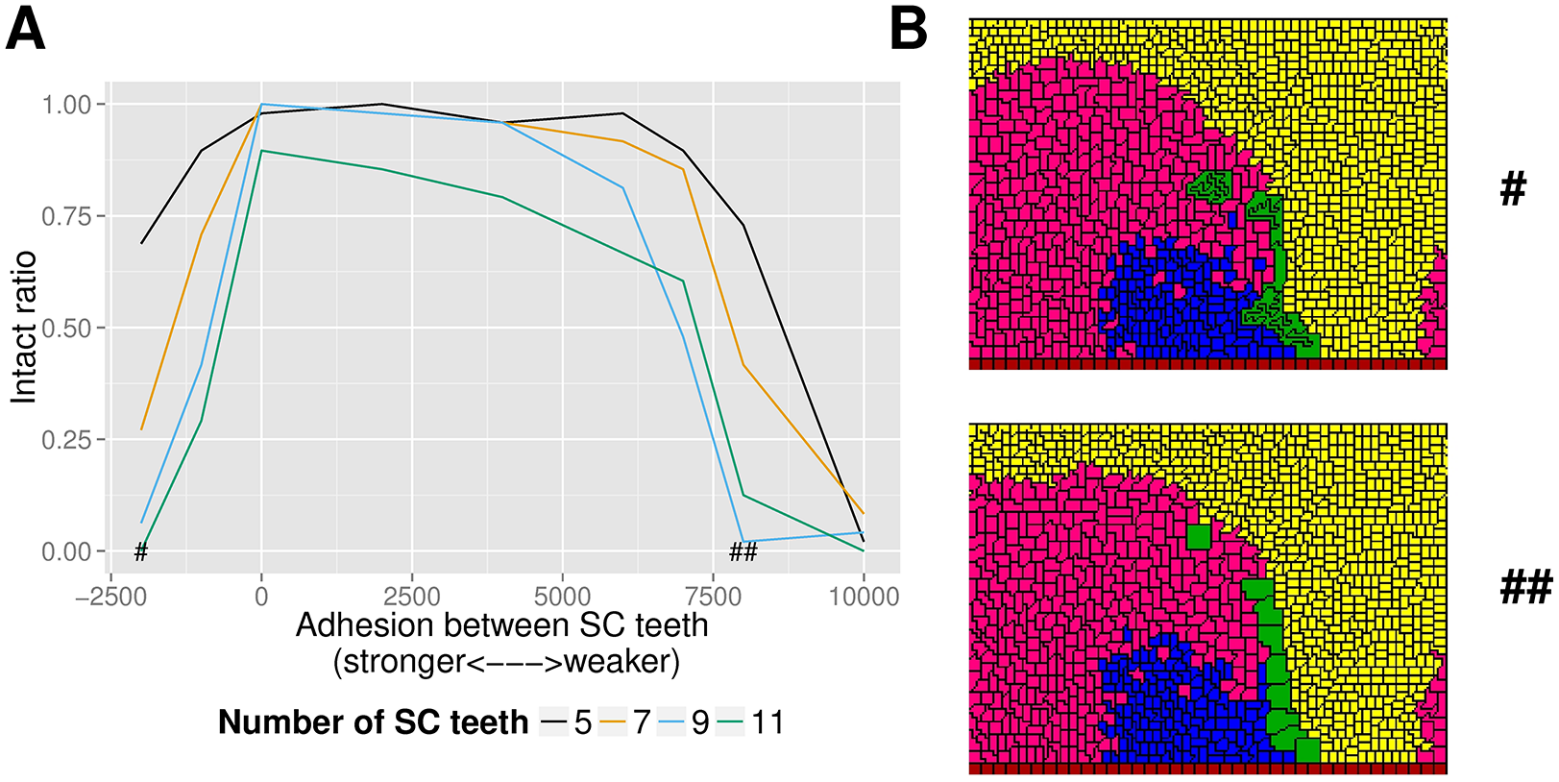
SC breaking statistics for different SC lengths and adhesion parameters. **A** Graph of intact ratio vs. adhesion parameter between SC teeth for rotated SC simulations of various SC lengths. **B** Two different parameter regimes for SC breaking. Top: SC breaking in the parameter regime where mutual adhesion between SC teeth is strong. This 9-tooth example is from the adhesion parameter regime marked with “#” in **A**. Bottom: SC breaking in the parameter regime where mutual adhesion between SC teeth is weak. This 9-tooth example is from the adhesion parameter regime marked with “##” in **A**.

In Fig 6A we show the statistics of SC breaking during rotation of the 36 sets of simulations covering all four SC lengths and a range of adhesion parameters between SC teeth. An SC can be classified as “intact” or “broken” based on whether a continuous path can be traced across every SC cell without trespassing on other types of cells. The “intact ratio” on the y-axis is the ratio of the number of intact rotated SCs to the total number of simulations (48) in a set. Therefore, an intact ratio of 1 means all 48 rotated SCs are intact for that set of simulation and 0 means every rotated SC is broken. Overall, simulated SCs break more easily during rotation as their length (number of SC teeth) is increased, irrespective of adhesion parameters between SC cells. For shorter 5-tooth SCs, intact ratio is virtually 1 across most examined adhesion parameters. However, for longer 9 or 11-tooth SCs, intact ratio drops to 0 for some adhesion parameters. Especially for 11-tooth SCs, intact ratio is in general no higher than 0.9 across all examined adhesion parameters *J*(*SCT*, *SCT*) (Table 3 and Eq 2).

**Table 3:** Adhesion parameter *J* between two cell types (see Eq 2), unless otherwise specified. A value of “S” indicates that the parameter is dependent on specific simulations. *J* is symmetric against interchange of the two cell types.

EP1	EP2	EP3	SCT	cell type *c*
1000	1000	1000	10000	EP1
	1000	1000	10000	EP2
		1000	10000	EP3
			S	SCT

As a function of adhesion parameters between SC teeth (x-axis), SCs break less often during rotation when adhesion between SC teeth is moderate. When adhesion between SC teeth becomes too weak, cells of another type more easily intrude into the space between successive SC teeth during rotation, thus breaking the comb. Paradoxically, when adhesion between SC teeth becomes very strong, SCs are also more prone to breaking. In this parameter regime, the SC teeth maximize surface contact with one another and form clusters, sometimes more than one (thus breaking the comb). The reason behind such maximization of surface contact is because the system is encouraged for doing so by virtue of a negative adhesion parameter. Thus, the system may find the rewards of maximization of surface contact outweigh the penalties imposed on altering the shape of SC teeth. The formation of clusters impedes the push from the expanding distal epithelial cells. As a result, many of the rotated SCs in this parameter regime are badly malformed, even if these SCs may be intact at the conclusion of the simulation. Fig 6B shows two examples of SC breaking, with the top one from the strong adhesion regime and the bottom one from the weak adhesion regime. Comparison between these two examples reveals qualitative differences between SC breakings in the two parameter regimes, in that serious malformation of SCs is also present in the strong adhesion regime but not so in the weak adhesion regime. (Every SC breaking example from Fig 5B is from the weaker adhesion regime. These examples are thus more qualitatively similar to the bottom panel of Fig 6B.)

#### SC length and adhesion on the ABASCT statistics of rotated SCs

**Fig 7:**
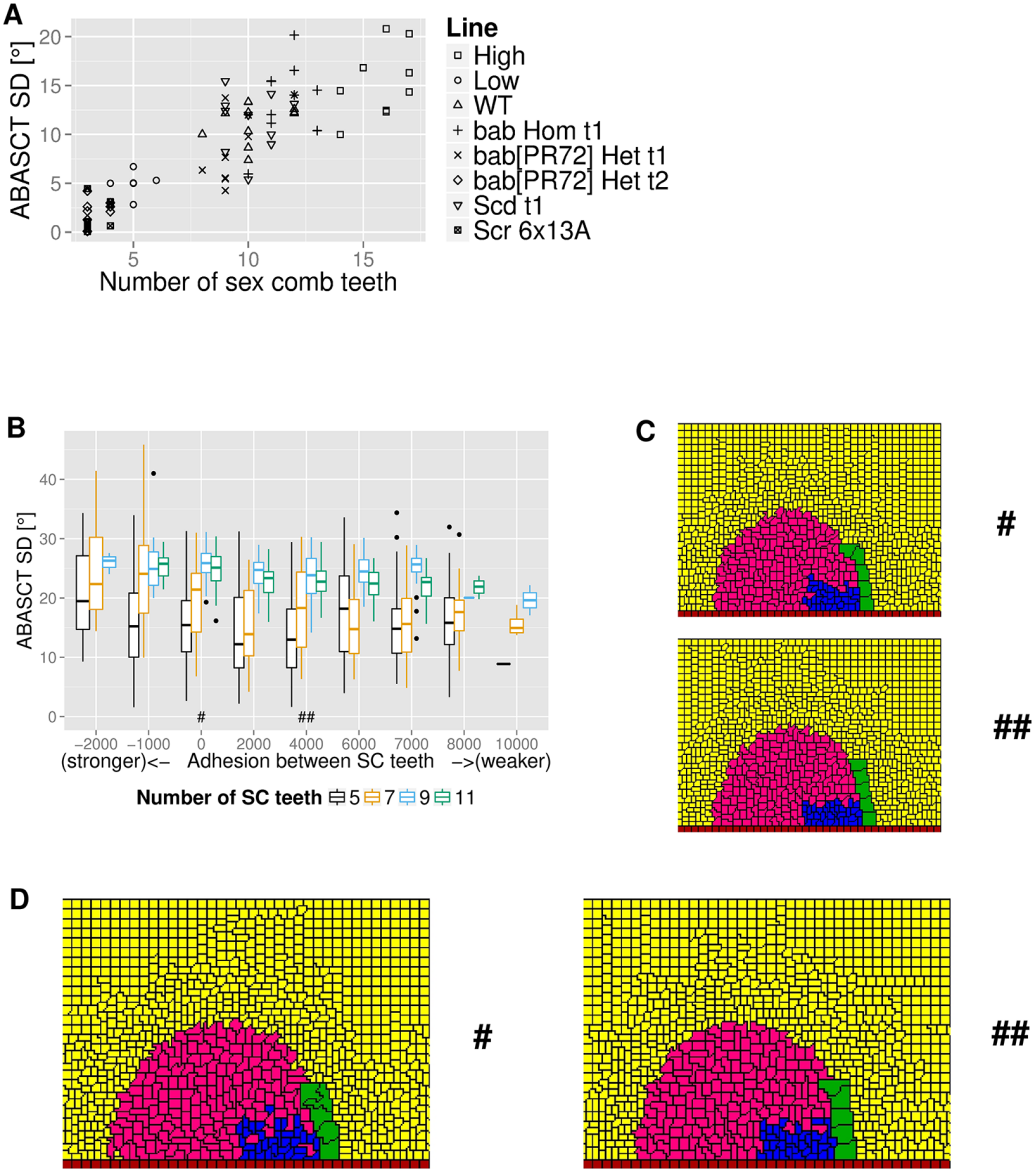
Statistics on ABASCT standard deviation for both experiment and simulated SC rotations. **A** Graph of ABASCT SD vs. SC length in experiments. Please see Table 4 for details on the mutant genotypes studied. **B** Graph of aggregate ABASCT SD statistics of simulated and intact rotated SCs, grouped by adhesion parameters, of all SC lengths examined. **C** Example outputs showing interplay between adhesion and expansion of epithelial cells on ABASCT SD. Upper panel. Higher ABASCT SD is generally obtained in rotated SCs with stronger adhesion between SC teeth. This 5-tooth example is drawn from the adhesion parameter regime marked with “#” in **B**. Lower panel. Lower ABASCT SD is generally obtained in rotated SCs with weaker adhesion between SC teeth. This 5-tooth example is drawn from the adhesion parameter regimes marked with “##” in **B**. **D** Example outputs showing “slowed down” rotation of 5-tooth SCs. With the exception of a larger *τ* for epithelial cell expansion, the example from the left panel shares identical parameters with the top one in **C** (adhesion parameter regime marked with “#”), while the example from the right panel has the same parameters as the bottom one in **C** (adhesion parameter regime marked with “##”).

To test the effect of changing the number of teeth on SC shape, we studied *D. melanogaster* mutations and artificially selected lines with variable comb lengths. Analysis of adult legs showed a departure from linearity of rotated SCs as the length of SC is increased. While short combs tend to be straight, long combs tend to bend. Such a departure from linearity is reflected in the ABASCT SD statistics of rotated combs (Fig 7A). Fig 7B shows the results of the statistics of ABASCT SD of simulated intact rotated SCs, grouped by the strength of adhesion between SC teeth (x-axis). In every adhesion parameter examined, we observe that longer SCs are generally, although not always, associated with a higher ABASCT SD median (horizontal line inside every coloured bar is the location of the median, the height of the entire bar not including the whiskers captures approximately half of data, the upper (lower) extent of whisker captures data up to 1.5 times the interquartile range from the 75th (25th) percentile; also see Figure C in S1 Text for histograms of ABASCT SDs for all intact SCs; Figure D in S1 Text for the presentation of the same ABASCT SD results with mean ±1SEM values; Figure E in S1 Text and Table C in S1 Text for results of statistical analyses). Furthermore, for each SC length examined, the ABASCT SD median decreases in general as adhesion between SC teeth becomes weaker (i.e. adhesion parameters getting more positive), up to some optimal adhesion before ABASCT SD median increases again. It means that there is an increased occurrence of straighter rotated SCs at some optimal adhesion parameters. Fig 7C illustrates the observation with a 5-tooth comb. These two simulations from the top and bottom panels share the same seed for pseudo random number generation but different adhesion parameters between SC teeth, with the top one having parameter 0 (stronger adhesion) and the bottom one having parameter 4000 (weaker adhesion). It is obvious that the rotated SC example from the weaker adhesion regime is more linear than the more varied shape of the one from the stronger adhesion regime. In fact, the ABASCT SD value for the top comb is about 22° while the one from the bottom is only about 3°.

**Table 4:** Summary of mutant fly genotypes studied.

Fly genotype	Abbreviation	Tarsal segment studied	Source
*Sex comb reduced*^6^/ *Sex comb reduced*^13^	Scr^6^/Scr^13^	First leg, first tarsal segment.	Anthony Percival-Smith lab
*bric à brac*^*PR*72^	*bab*^*PR*72^	First leg, first (t1) and second (t2) tarsal segments in both homozygous (Hom) and heterozygous (Het) flies.	Godt lab [33]
*Sex comb distal*^1^	*Scd*^1^	First leg, first tarsal segment.	Bloomington 5070

#### Rate of expansion of epithelial cells partially explains the difference in ABASCT SD statistics between longer and shorter SCs

Why is a more linear rotated SC favoured when adhesion is moderate or the SC is shorter? One possible mechanism may be the interplay between epithelial cell expansion (which drives SC rotation) and mutual adhesion between SC teeth (which to a certain extent impedes rotation–see Fig 6B). In experiments, the time taken for the SC to complete rotation is positively correlated to the length of SCs. For example, a 5-tooth SC takes roughly 12 hours to complete rotation, while an 11-tooth SC takes roughly 26 hours [15]. In our simulations, such information is primarily encapsulated in the values of time constants of expansion of epithelial cells *τ*(*c*(*σ*)) (Eq 5), with a higher value of time constant representing a longer time taken for the distal cells to asymptotically reach $a_{target}^{term}$ (Table 2). Thus, everything else being equal, a larger time constant for distal cell expansion means a slower expansion rate and a longer time for SC rotation to complete. (Alternatively, a smaller $a_{target}^{term}$ parameter also means a slower expansion rate where the cells eventually expand to reach a smaller terminal cell size, but this has little effect on the time the cells take to reach the terminal size.) Given such a large disparity of rotation times, we ask whether the speed of rotation could be an underlying factor behind the ABASCT SD statistics of Fig 7C. Although manipulation of rotation speed is difficult experimentally, it is relatively easy in simulations. In these extra simulations, we artificially “slow down” the rotation in 5-tooth SC simulations by increasing *τ* to match the one in 11-tooth SC simulations (see Table 2).

Fig 7D shows two example simulation results of these “slowed down” 5-tooth rotations. In both “slowed down” simulations, we observe more malformation of SCs, in particular with the SC teeth at the tip region. For the one on the left (stronger adhesion between SC teeth), the malformation is more serious in that the top two SC teeth are fused horizontally. The example on the right (weaker adhesion between SC teeth) displays a slight “cane” shaped rotated SC where the topmost SC tooth does not appear to receive sufficient push to reach to the top. As a result of such malformations of the “slowed down” 5-tooth simulations, we observe generally increasing median and a considerably broader distribution of ABASCT SDs as compared to the original “faster” 5-tooth simulations (Figure F in S1 Text).

The reason that a “slowed down” epithelial cell expansion increases malformation of 5-tooth SCs is because the balance between cell expansion rate and adhesion between SC teeth is disrupted. Although increased instances of intact SCs are observed, a slower expansion of epithelial cells reduces the push to SC teeth, particularly in the ones closer to the tip which are more sensitive to the amount of push than the ones closer to the base. Therefore, the strength of SC adhesion prevails in impeding the push, creating malformation.

### A suitable temporal sequence for the expansion of distal epithelial cells during SC rotation partially rescues long SCs from breaking

**Fig 8:**
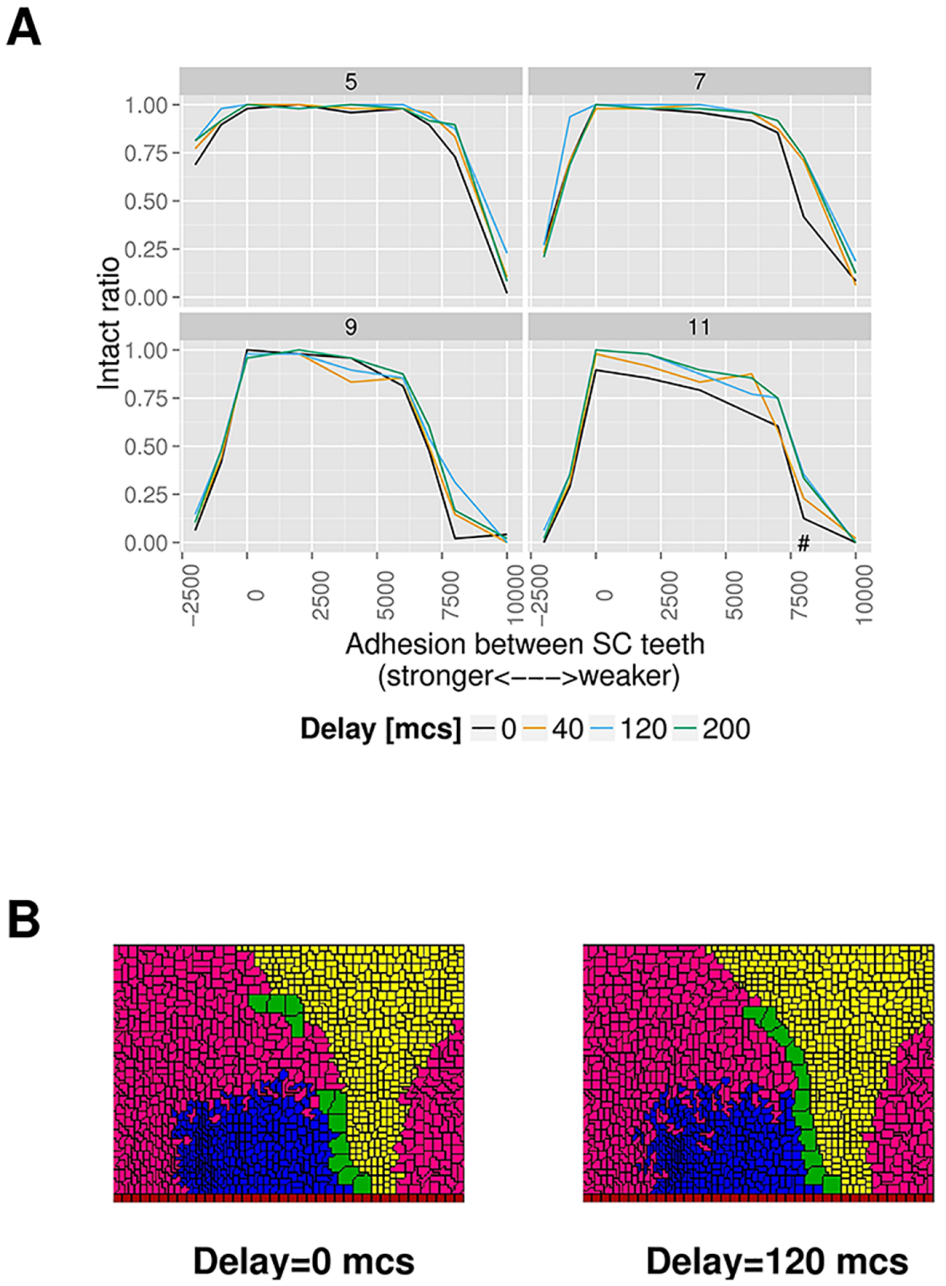
A suitable temporal sequence of expansion of distal epithelial cells improves breaking statistics for longer SCs. **A** Four graphs of intact ratio vs adhesion between SC teeth, with different line colours representing the effect of different magnitudes (in mcs) of delayed expansion of the distal epithelial cells closer to the base of the SC (blue coloured EP1 cells in Fig 5B) relative to the epithelial cells closer to the tip of the SC (magenta EP2 cells in Fig 5B) on intact ratio. Each graph shows the results for one particular SC length (5, 7, 9 or 11-tooth SC). **B** Two example 11-tooth SC simulations showing that delayed expansion of distal epithelial cells closer to the base of SC reduces incidences of SC breaking during rotation. Left: all distal epithelial cells expand at the same time. Right: delayed expansion of distal epithelial cells closer to the base of SC (i.e. EP1) by 120 mcs. Both example simulations share the same random seed and other parameters (with SC adhesion in the parameter regime labelled with “#” in **A**) except the cell expansion sequence. S3 and S4 Videos show the frame-by-frame capture of the two simulations, respectively.

Hitherto we have mostly discussed how spatial properties of distal epithelial cells may help proper SC rotation. However, even with inhomogeneous spatial patterning of distal cells, the rotated simulated SCs suffer from a decreasing intact ratio as the length of SC is increased (Fig 6A). In particular, the intact ratio of 11-tooth SCs drops considerably across virtually all examined SC adhesion parameters when compared with the shorter 9-tooth SCs. In this section we explore whether introducing temporal dynamics to the expansion of distal epithelial cells, in addition to spatial patterning, could improve the breaking statistics of rotated SCs. We perform additional simulations in which the blue distal cells (EP1) expand later in the simulations, while keeping everything else identical. There are 3 × 36 = 108 additional sets of simulations, which cover 3 magnitudes of delayed expansion of EP1 cells (40, 120 and 200 mcs). Visual examination of the aggregate statistics suggests that these extra simulations share similar *α* and ABASCT SD distributions with the regular, non-delayed simulations (Figure G in S1 Text and Figure H in S1 Text). However, comparison of intact ratios between the delayed and non-delayed simulations (Fig 8A) shows that while there is no apparent improvement on intact ratios for shorter 5,7,9-tooth SCs, there is a consistent improvement for 11-tooth SCs across almost all SC adhesion parameters (Table D in S1 Text). In fact, with an appropriate delay parameter (∼ 120 mcs), intact ratios of 11-tooth SC approach that of a 9-tooth one. Too high a delay parameter (> 150 mcs) does not make intact ratios higher. On the contrary, it causes compression of cells at the centre of the distal region, a phenomenon not readily observed in experiments. Fig 8B shows two examples of 11-tooth SC simulations in which a delayed expansion of EP1 affects the final outcome of the SC, with the one having a delayed expansion of EP1 enjoying an intact SC while the non-delayed simulation has a broken rotated SC.

**Fig 9:**
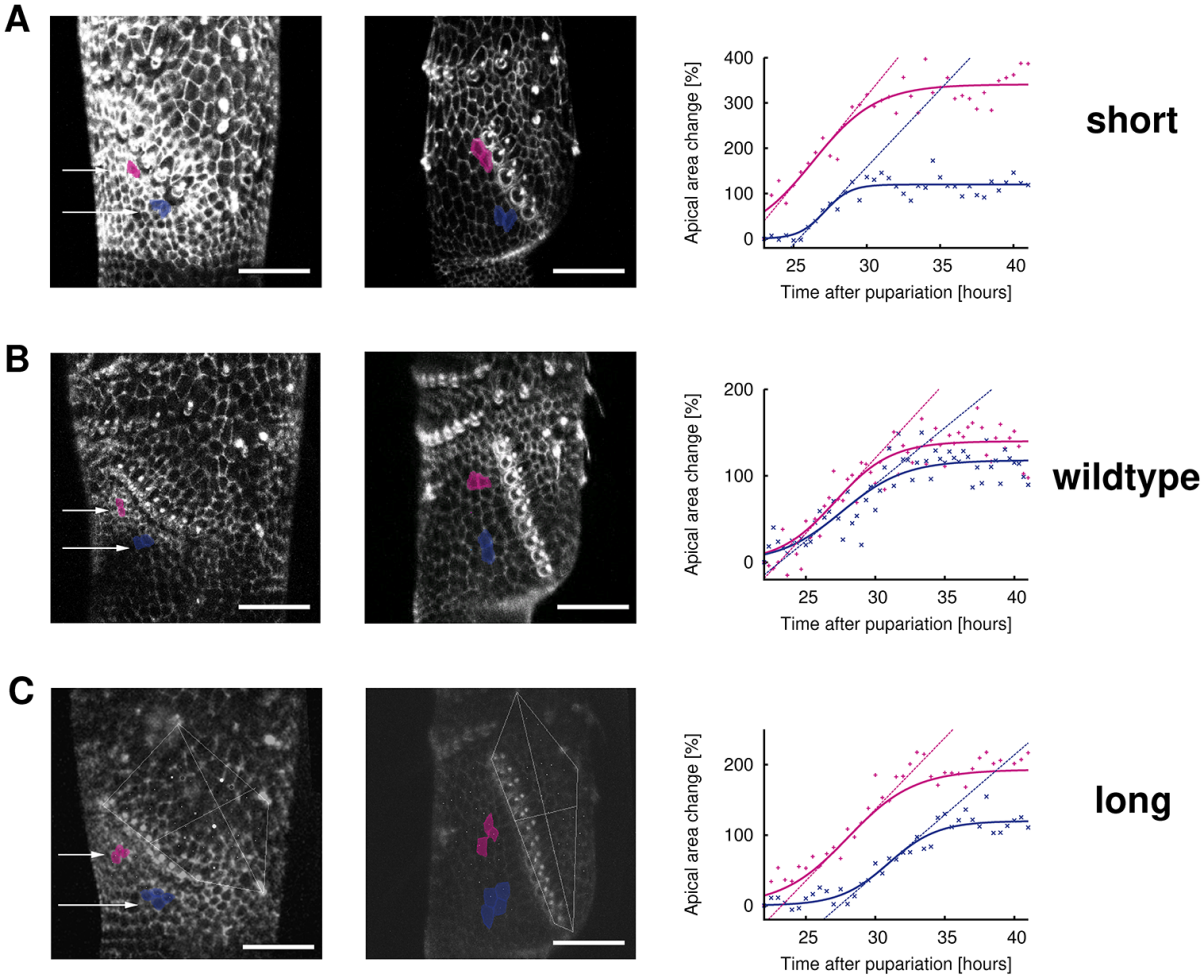
Experiments showing rotation of longer SCs exhibits delayed expansion of distal epithelial cells closer to the base of comb. **A** Left: confocal image of short SC and its surrounding cells at the start of area measurement (23 hours AP). Shaded areas (magenta and blue) are the cells selected for area measurement. Middle: same confocal image with identical shaded cells at 36 hours AP (end of area measurement at 41.5 hours AP). Right: graph of percentage change of apical areas of the shaded cells vs. time and the fitted logistic curves. Each coloured straight line is the tangent at the inflexion point of the same coloured logistic curve. Its intercept with the time axis is mathematically constructed as the start of the expansion phase of the cells. Each scale bar: 20 *μ*m. **B** Same as **A** but for wildtype comb. Start of area measurement at 22 hours AP. **C** Same as **B** but for longer comb. Start of area measurement at 22 hours AP.

To determine whether such a temporal delay exists in experiments, we collect data on the time course of growth of distal cells in each of our short, wildtype and long SC examples (Fig 9). The percentage growth in area of these cells (as compared to the start of measurement at either 22 or 23 hours AP) is then fitted with logistic functions to determine the time lag between the expansion phase of distal epithelial cells in different areas. The results of curve fitting suggest that, in our long SC example (Fig 9C), there is a delay in the expansion phase of distal epithelial cells closer to the base of the SC relative to the distal epithelial cells closer to the SC tip, with the start of expansion phase of magenta cells (closer to the SC tip) at 23.27 hours (22.25-24.28 hours 95% CI), while that of blue cells (closer to the SC base) is 27.42 hours (26.34-28.51 hours 95% CI). In the wildtype example (Fig 9B), however, there is no statistically significant difference between the start of the expansion phase of the selected magenta and blue cells. Our experimental results provide evidence that longer SCs may take advantage of the time delay to achieve proper rotations. We emphasize that even though delayed expansion of blue coloured cells is also detected in our short SC example (Fig 9A), it does not represent an inconsistency between modelling and experiments because delayed expansion of EP1 (blue) cells does not statistically affect the intact ratio of shorter SCs (Fig 8A).

## Discussion

### Implications of results to evolution and development

SC rotation in *D. melanogaster* involves a complex pattern of cell behaviours (such as cell proliferation, shape change and movement) [15, 27, 34, 35]. However, how these multiple cellular processes contribute to SC morphogenesis has remained elusive. We wished to understand how the temporal and spatial properties of each cell behaviour contribute to normal comb rotation. Although this would be experimentally impractical, it is possible to explore these properties computationally by simulating the effects of varying cell behaviours. From extensive simulations, we deduce that proper rotation of the SC depends on precise spatio-temporal dynamics of distal cells. The initial spatial cell size distribution in the distal epithelium was found to be important to minimize SC bending and malformation. In particular, distal cells closer to the SC base (pivot point) have larger initial apical areas than cells farther away from the base. Such inhomogeneity in initial apical areas of distal cells translates into a differential push across the length of SC as the cells expand, ensuring proper SC rotation in simulations. In addition to spatial distribution, a temporal component was also discovered in our simulations, when delay in expansion of distal cells closer to SC base relative to cells closer to the SC tip reduces incidence of long SC breaking during rotation. These simulations are not only useful in showing that cell behaviours distal to the rotating comb are likely sufficient to provide the motive force for comb rotation, they also suggest relationships of which importance was not previously realized. In fact, having found in simulations of the spatio-temporal dynamics of the distal cells predicted to be crucial for proper SC rotations, we were able to go back to the original data in the 4D movies and confirm that this was indeed occurring biologically.

Not only did the simulations produce testable hypotheses concerning developmental mechanisms in the model species *D. melanogaster* they also suggested hypotheses concerning how changes in developmental processes might produce different SC patterns during fly evolution. For instance, changes in rotation angle might be due to changes in expansion of distal cells (Fig 4). Our work also suggests potential avenues for these changes and which paths might be forbidden. As an example, rotation of long SCs in our simulations are more difficult because increasing comb length generates multiple problems for movement including comb breaking, atypical alignment, among others (Fig 6). These results are consistent with the differing strategies to achieve SC morphogenesis taken up by multiple *Drosophila* species with long SCs. Most of the SCs from these species lack rotation, appearing already in a vertical position early in the developmental process [6, 9, 13]. Furthermore, other species with long rotating SCs such as *Drosophila guanche* display frequent broken and misaligned bristles [6], consistent with our simulations.

From our biological *Drosophila* experiments, we observed a relatively consistent linear shape (quantified by ABASCT SDs, [8, 13, 27]) of rotating SCs amongst *Drosophila* species, yet the orientation of SCs (i.e. *α*) has a larger variation [13]. On the other hand, we have discovered from our simulations that SC shape is primarily (though not exclusively) governed by the initial inhomogeneity of distal cell sizes (Fig 2), while SC orientation can be controlled by the subsequent rate of expansion of these cells (Fig 3). Therefore, if these two phenotypes (shape and orientation of SCs) follow distinct evolutionary trajectories, our simulation results suggest that there may be signatures left by evolution on the two corresponding governing cellular characteristics (initial inhomogeneity in distal cell sizes and subsequent expansion of these cells, respectively). In other words, a canalized SC shape implies a conserved spatial inhomogeneity in initial distal cell areas, but variation in SC orientation implies diversity in subsequent expansion rates of these cells. Preliminary evidence from Fig 4 suggests this is indeed the case. Summary statistics Fig 4D and subsequent statistical analyses in particular from Figure A in S1 Text show an approximate, albeit imperfect conserved existence of inhomogeneity of *initial* distal cell areas across the male fly genotype examples (both wildtype and mutant), which exhibit different SC orientations but similar SC shapes. It is primarily the variation in the subsequent expansion of epithelial cells between the fly genotypes that determines the SC orientation. Our results suggest the possibility of separate molecular mechanisms, which are born out of evolution and are continuously modified by it, underlying these two cell characteristics. In addition, our results raise the question of whether *Drosophila* species using developmental mechanisms other than rotation also canalize their SC shape while exhibiting higher variation in comb orientation.

### Future considerations

There are several shortcomings in this first work to study SC rotation *in silico* which warrant future improvements. First, although we attempt to replicate as close to experimental results as possible, *α*s in our simulations generally have slightly lower values than expected for normal rotation. Our results thus suggest that other cellular processes may also contribute to SC rotation. For example, joint formation is not included in our simulations yet previous studies have pointed out that it affects the basal part of SC [13] and constitutes the male-specific movement of sensillum campaniforme [15]. Effects of joint formation on SC rotation can be studied using photo-activated lines that can disrupt development precisely [36, 37].

We also did not systematically investigate the possibility of the “pulling” effect from proximal cells during SC rotation. A primary reason we focused on the distal, not proximal cells is that even without significant contraction in apical areas in the proximal region, proper SC rotation could still occur [15]. There is also an absence of experimental observation of any initial spatial arrangement of proximal cells similar to, but opposite in direction of, distal cells. The apparent absence of such spatial arrangement means that proximal cells are unlikely to exert a coordinated pulling force similar to the pushing force of distal cells during SC rotation. Moreover, we did not observe from experiments a clear spatial or temporal sequence in which proximal cells extrude from the epithelium, meaning that it is more likely that the proximal cells were passively “squeezed” due to external pressure rather than actively contracting generating a pulling force. Nevertheless, for future considerations it would be desirable to quantify the effects (if any) of proximal cells in next iterations of simulation models. Currently, although cell extrusion is included in our simulations, its effects are not examined thoroughly. While cell extrusion is shown to be the consequence of cell crowding rather than a major driving force of development [38, 39], future simulations could better quantify its effects above the SC and test whether it buffers comb shape.

From the simulation perspective, the parameters used here are chosen to reproduce SC dynamics as observed in experiments. A consequence of such an approach is that these parameters may not correspond to the mechanical characteristics of actual SC or epithelial cells. In theory, mechanical characteristics of cells are related to the spatial derivatives of the Hamiltonian *H*_*eff*_ used in CPM. However, the exact correspondence is usually complicated and may depend on the specific topology of the cellular systems studied. Although there have been attempts to perform CPM simulations with parameters based on matching the terms of *H*_*eff*_ and the mechanical characteristics of cells [40], the majority of simulation examples in the literature use our approach of choosing parameters based on the correct reproduction of observed cellular behaviours [41]. We expect the major conclusions established in this work should be largely independent of the specific parameters used in the simulation model, so long as those parameters also demonstrate observed cellular behaviours. For example, it would be extremely difficult to imagine something as fundamental as the spatio-temporal properties of distal cells not to hold if another set of more “realistic” parameters were used for simulations, in particular when these previously unnoticed spatio-temporal properties are themselves demonstrated in experiments (Figs 4 and 9). Future simulations could address this issue by further refining the parameters so as to reflect the actual mechanical characteristics of cells.

In addition, some of the cells in the simulations have a very small initial lattice size (≤ 5 pixels). This could potentially create issues when the stochastic effects of the simulations become large. For example, these small cells could disappear due to stochastic effects early in the simulations with no chance to expand. To check whether our simulation results are scalable in size, we performed some sample simulations with identical cell arrangements but with a resolution 4 times as much (i.e. 228 × 168 pixels in the sample simulations vs. 114 × 84 pixels–Figure I in S1 Text). We discovered that SCs were able to properly rotate just as in the original simulations. Thus, although some anomalous effects could be present in the simulated cells in the original simulations due to their smaller grid sizes, these are likely not severe enough to have impacted the conclusions of this work.

In the area analysis of Fig 4 and Table A in S1 Text, we classified distal cells by a demarcation line drawn at the halfway length mark of the SC on every confocal image where area analysis was performed. The demarcation line is horizontal for 30° ≤ *α* ≤ 90° but perpendicular to the SC for *α* < 30°. Any distal cell above the line (closer to SC tip) was defined as EP2 and any distal cell below it (closer to the SC base) EP1. Such an approximate method of classifying distal cells in experiment could be problematic because movement of cells during SC rotation means that some of the distal cells may have different classifications at the start and at the end of SC rotation. To ensure that our classification method is robust, we have performed individual cell tracking on a sample fly leg (Figure J in S1 Text). The advantage of individual cell tracking is that the cell labels (whether the cells are defined as EP1 or EP2) are consistent throughout the entire area analysis. We obtained identical conclusions with individual cell tracking as with our original method, thus showing that our approximate method of cell classification does not significantly affect the conclusions established in this work.

Separately, a certain degree of cell compression was observed along the EP1/EP2 boundary in the simulations of Fig 3A and 3B, while no such simulation artefact was observed in Fig 3C. Cell compression occurs in some simulations because of the inhomogeneous expansion rates amongst the distal cells due to their different initial areas, terminal target areas and timing of expansion. Therefore, some EP1 or EP2 cells are receiving more pushing forces than others, causing compression. In particular, the imbalance in pressure along the EP1/EP2 boundary is more acute in Fig 3A and 3B simply because we reduced the expansion of magenta EP2 cells in Fig 3A and 3B (as compared to Fig 3C), but we did not reduce as much expansion for the blue EP1 cells. We believe that such cell compression can be further mitigated by fine-tuning initial distal cell area configurations, terminal target areas and λ parameters.

As well as expansion, distal cells were observed experimentally to elongate along the y or the ordinate axis during SC rotation. While we have included cell polarization (*ϵ* in Table 2) in our model, it remains an interesting question the extent of distal cell polarization contributes to SC rotation. Although we have not systematically examined the issue from a simulation standpoint, in the early model development we ran limited sets of simulations in which distal cell expansion was isotropic (i.e. cell polarization was absent), we noticed that simulated SCs could still properly rotate but the rotation angle (*α*) was generally smaller by 10-15° as compared to simulations with cell polarization. More extensive simulations are required to fully elucidate the relationship between cell polarization and SC rotation.

Finally, during the measurement of temporal changes in apical areas of distal cells (Fig 9), we noticed that in addition to the primary growth pattern following logistic function (fitted curves of Fig 9A, 9B and 9C), there also exists oscillatory behaviours for several of the cell samples measured, in particular the ones in Fig 9A and 9C. Although actin-myosin binding dynamics are thought to be responsible for similar oscillatory behaviours with a much shorter time scale [42], we remain agnostic about the possible mechanisms for cell area oscillations in our case because of their long periods (up to a few hours). Future experiments are required to answer the open question about the mechanism underlying these oscillations and whether such oscillatory behaviours contribute to SC rotation.

## Materials and Methods

### Simulations

#### Cellular Potts Model

The two-dimensional CPM [43–45] is used to simulate SC rotation. The simulated area is partitioned into *n*_*x*_ × *n*_*y*_ unit square pixels. A “cell” is defined to occupy one or more pixels in a contiguous manner. Each pixel can only be at one time occupied by a single “cell”. Fig 1G shows a hypothetical configuration of a simulated area and also illustrates the relationship between a pixel and a cell (Fig 1H is a blow-up of a portion of Fig 1G). Given initial (i.e. which pixel belongs to which cell at *t* = 0) and boundary conditions, the subsequent development of cells is governed by an effective Hamiltonian (energy) *H*_*eff*_. Briefly, with an externally supplied “temperature” parameter *T*, the simulated system obeys the Boltzmann distribution with the probability of the system having a particular energy configuration proportional to $\text{exp}(-\frac{H_{eff}}{T})$.

Our effective Hamiltonian has three terms,

$$\begin{array}{ccc} H_{eff} & = & H_{boundary}+H_{length}+H_{area}. \end{array}$$ (1)

The three terms on the right hand side of Eq 1 represent the adhesion between cells, the tendency of the cell to change shape, and the tendency of the cell to become larger or smaller, respectively. Mathematically, let $\vec{v}\equiv \left(x,y\right);\;x,y\in N$ represent the unique identity of each pixel in the simulation area (for example, (0, 0) identifies the bottom-left most pixel in the left panel of Fig 1G, and (0, 1) the one directly on top of it), $\sigma (\vec{v})$ represent the corresponding “cell” index, and $c(\sigma \left(\vec{v}\right))$ represent the *type* of the cell. *H*_*boundary*_ is defined as:

$$\begin{array}{ccc} H_{boundary} & = & \sum_{\begin{array}{c} \vec{i},\vec{j}\\ \vec{j}\in N\left(\vec{i}\right) \end{array}}J\left(c\left(\sigma \left(\vec{i}\right)\right),c\left(\sigma \left(\vec{j}\right)\right)\right)\times \left(1-\delta_{\sigma \left(\vec{i}\right),\sigma \left(\vec{j}\right)}\right), \end{array}$$ (2)

where *J* is the adhesion parameter between two cell types, $\delta_{\sigma \left(\vec{i}\right),\sigma \left(\vec{j}\right)}$ is the Kronecker delta function which prohibits summation of energies of pixels within the same cell. $N(\vec{i})$ is $\vec{i}$’s neighbourhood. The neighbourhood $N(\vec{i})$ for contact energy is defined as the 4 closest pixels surrounding $\vec{i}$. Heuristically, the higher the numerical value of *J* between the two cell types, the more difficult it is for these two cell types to “clump” together.

*H*_*length*_ has the form,

$$\begin{array}{ccc} H_{length} & = & \sum_{\sigma }\lambda_{l}\left(c\left(\sigma \right)\right)\times {\left(l\left(\sigma \right)-l_{target}\left(c\left(\sigma \right)\right)\right)}^{2}. \end{array}$$ (3)

*H*_*length*_ is summed over all cell indices, not pixels as in Eq 2. *l*(*σ*) represents the current length of a cell along the elongation axis, and *l*_*target*_ is the target length value parameter. This target length value parameter can vary from one cell type to another (Table 2). Essentially, a penalty is imposed whenever the perimeter of a cell deviates from the target length value. The severity of the penalty is partially determined by the value of λ_*l*_.

Finally, *H*_*area*_ has a similar form as *H*_*length*_,

$$\begin{array}{ccc} H_{area} & = & \sum_{\sigma }\lambda_{a}\left(\sigma \right)\times {\left(a\left(\sigma \right)-a_{target}\left(c\left(\sigma \right)\right)\right)}^{2}. \end{array}$$ (4)

#### The Metropolis algorithm

The time evolution of the simulated “cells” in the system is characterized by attempted pixel “flips” from neighbouring “cells”. The Metropolis algorithm [46] is used to carry out such pixel “flips” at each Monte Carlo Step (mcs). During each mcs, a fixed number of pixels (“target pixels”) are chosen randomly from the system for attempts to have their “cell” indices changed to one of its neighbouring “cells”. For each target pixel, a neighbouring pixel is also randomly chosen. The size of the “neighbourhood” for flip attempts is determined by the neighbour order parameter in Table 1. A neighbour order of 2 means that the neighbourhood consists of the 8 surrounding pixels closest to the target pixel. If the target pixel belongs to the same “cell” as the chosen neighbouring pixel, no flipping of cell index of pixel will occur. However, if the neighbouring pixel is from a different “cell”, the simulation proceeds to calculate the effective energy *assuming the target pixel is flipped to belong to the same “cell” as the neighbouring pixel*. We denote this effective energy as $H_{eff}^{1}$, and the original effective energy without the assumed index flip as $H_{eff}^{0}$. If $H_{eff}^{1}<H_{eff}^{0}$, the assumed flip will be realized with certainty. If $H_{eff}^{1}\geq H_{eff}^{0}$, the assumed index flip will be realized with probability $\text{exp}(\frac{H_{eff}^{0}-H_{eff}^{1}}{T})$. One can regard this attempted index flip as an “invasion” by neighbour pixels onto the target pixels. The successful “defence” of the target pixels depends on the relative values of $H_{eff}^{0}$ and $H_{eff}^{1}$, and also the stochastic factor governed by the “temperature” parameter *T*. These attempted index flips are the only way “cells” can change their sizes, shapes and locations.

#### Initial cell configuration and boundary conditions

Fig 1I shows an example initial cell configuration of 9-tooth SCs. Unless otherwise specified, in all simulations (and in Fig 1I) the entire simulation area consists of *n*_*x*_ × *n*_*y*_ = 114 × 84 = 9576 pixels. We use 3 types of epithelial cells (EP1, EP2, EP3), one type of cells for sex comb teeth (SCT) and one type of cells for the “barrier” (BA). In this Fig 1I and all other figures depicting cell configurations, we use blue for EP1, magenta for EP2, yellow for EP3, green for SCT and red for BA. We define “distal” as the region below the SCT and “proximal” as the region above. Therefore, EP1 and EP2 are sometimes referred to as “distal epithelial cells” (or simply “distal cells”) and EP3 is referred to as “proximal epithelial cells” (or “proximal cells”). To take into account the cylindrical shape of the forelegs of the *Drosophila* species, we impose the periodic boundary condition at the “left” and “right” boundaries (i.e. when *x* = 0 and when *x* = 113). The default Neumann no-flux boundary condition is used at the “top” boundary (i.e. when *y* = 83), whereas the existence of the infinitely rigid “barrier cells” at the bottom creates an absorbing boundary condition there.

#### Additional customizations

Our computational model is further customized to simulate behaviours specific to SC rotation.

*Change in areas of epithelial cells.* If we leave *a*_*target*_ as a constant parameter, cell sizes only fluctuate around the value of *a*_*target*_ specified for each cell type. To simulate the significant expansion or dropping in sizes of some cells in experiments, we periodically update *a*_*target*_ of the simulated epithelial cells, with the aim that the target areas also evolve asymptotically in time to a terminal value, as follows.

$$\begin{array}{c} \begin{array}{cc} a_{target}\left(c\left(\sigma \right),t+\Delta t\right) & =a_{target}\left(c\left(\sigma \right),t\right)\times \left(1+\left(\frac{a_{target}^{term}\left(c\left(\sigma \right)\right)}{a_{target}\left(c\left(\sigma \right),t\right)}-1\right)\times \frac{\Delta t}{\tau (c(\sigma ))}\right), \end{array} \end{array}$$ (5)

where $a_{target}^{term}$ is the value of terminal target area specific to each type of epithelial cell, *τ* is the time constant for the apical area expansion (contraction) of distal (proximal) cells, and Δ*t* (set to 10 mcs for all simulations) the time between two successive updates. Eq 5 is the first order approximation of the solution (Euler’s method, see for example [47–49]) to the following differential equation, with initial condition *a*_*target*_(*t* = 0) > 0,

$$\begin{array}{c} \begin{array}{cc} \frac{da_{target}\left(t\right)}{dt} & =\frac{a_{target}^{term}-a_{target}\left(t\right)}{\tau }. \end{array} \end{array}$$ (6)

Every update of *a*_*target*_ (Eq 5) also leads to a corresponding update of *l*_*target*_ with the new *l*_*target*_ to be set to the integer ceiling of square root of the updated *a*_*target*_ (Table 2).

In addition, to facilitate cell extrusion, we update *a*_*target*_ of individual EP3 based on current areas of those cells. Thus, we set in our model *a*_*target*_(*c* = *EP*3) to 0 and both λ_*area*_ and λ_*length*_ to some arbitrarily large positive values once their sizes drop to below 3 pixels.

*Directional preference in area expansion of epithelial cells.* Some epithelial cells in experiments have a preference to expand vertically. Therefore, we modify the components of *H*_*area*_ (Eq 4) to provide axial dependence to *H*_*area*_. The modified *H*_*area*_ becomes:

$$\begin{array}{c} \begin{array}{cc} H_{modarea} & =\sum_{\sigma^{{}^{\prime }}}\lambda_{a}\left(\sigma^{{}^{\prime }}\right)\times {\left(a\left(\sigma^{{}^{\prime }}\right)-a_{target}\left(c\left(\sigma^{{}^{\prime }}\right)\right)\right)}^{2}\\ & +\sum_{\sigma^{”}}\lambda_{a}\left(\sigma^{”}\right)\times {\left(a\left(\sigma^{”}\right)-a_{target}\left(c\left(\sigma^{”}\right)\right)\right)}^{2}\times (1-\epsilon \left(c\left(\sigma^{”}\right)\right)\\ & \times (1-\frac{1{\text{pixel}}^{\frac{1}{2}}\times {\text{sin}}^{2}\theta \left(\sigma^{”}\right)}{R(\sigma^{”})})). \end{array} \end{array}$$ (7)

*H*_*modarea*_ is calculated twice at each attempted pixel flip (see “The Metropolis algorithm.”). In Eq 7, $\sigma^{”}$ represents the subset of “cells” with these two characteristics: 1) belonging to any of cell types *c* which exhibit an axial preference to expand, and 2) one of the pixels belonging to those “cells” is chosen to be either the “target” or “invading” pixel during that attempted spin flip procedure. *σ*′ represents the rest of the “cells” in the simulated system. *ϵ* is the axial parameter that determines the strength of the axial preferences of the cells (*ϵ* = 0 represents isotropic expansion); *θ* and *R* are cell geometry variables which are illustrated graphically in Fig 1H. (To maintain the relative scale, $R\to \frac{R}{2}$ in Eq 7 for Figure I in S1 Text.)

#### Choices of parameters and implementing the simulations

All simulations were performed using compucell3d (version 3.7.5, [50]) on the WestGrid (www.westgrid.ca) computing facility. We implemented the additional customizations using Python to modify specific modules of compucell3d. Perl scripts were developed to generate initial cell configuration files in pif format. At regular time-steps and at the conclusion of simulation, output files in vtk (http://vtk.org) were stored for analyses. Tables 1, 2, 3 and 5 list the relevant parameters of different cell types and variables for simulations.

**Table 5:** Variables of the simulations.

Variable	Description	Units
*t*	time	Monte Carlo Step (mcs)
*x*, *y*	spatial coordinates, or pixel identifier	pixel^1/2^
*σ*(*x*, *y*, *t*)	Cell index	-
*c*(*σ*(*x*, *y*, *t*))	Cell type	-

### Post simulation data analyses

Unless otherwise specified, simulations were performed in sets. A set consists of 48 independent simulations. These 48 simulations share identical parameters and initial cell configurations, but differ in the seed for pseudo random number generation (Knuth’s subtractive method, [31, 51]) which affects the selection of pixels for attempted flips. Since these simulations can be regarded as multiple numerical experiments under the same parameters, we can extract attributes from these outputs and perform analyses to determine how different parameters affect outputs in a statistical manner. In other sections we have developed various metrics which quantify the well-formedness of the rotated SCs in simulations. These metrics were extracted from the final image output file of each simulation via Python with the NumPy package and APIs for vtk files. R (version 3.0.2, https://www.r-project.org, [52]) with the multcomp [53] and ggplot2 [54] packages was used for statistical analyses and visualization of results. Visualization of simulated SC rotations and data plotting were performed with paraview (version 4.0.1, www.paraview.org, [55]) and gnuplot (version 4.6, www.gnuplot.info) respectively. The grofit package of R [56] was used to fit cell expansion data of Fig 9, while gimp (version 2.8.10, www.gimp.org) was used for colour highlighting of cells there. The default Mersenne-Twister [57] in R was used as the pseudo random number generator for statistical analyses of Fig 4 (Table B in S1 Text).

### Experiments

#### Developmental measurements

Five genotypes were studied: ♂wt, ♀wt, the mutant heterozygous *bab^*PR*72^*, and males from the artificially selected lines for high and low number of SC teeth. High and low SC teeth number lines were developed by artificial selection for 24 generations, following which the lines were maintained by selecting every 4-5 generations [58]. While standard genetic crosses were sufficient to introgress the ubi-DE::cadGFP into the *bab^*PR*72^*, several generations of backcrossing were required to introgress the fluorescent marker into the artificially selected lines.

#### Morphological measurements for angle between adjacent sex comb teeth (ABASCT)

Forelegs of at least 20 individuals were used for each of the following *D. melanogaster* lines: wild type, low and high lines. Three mutants were studied: Scr^6^/Scr^13^, *bab*^*PR*72^ and *Scd*^1^ (Table 4).

#### Confocal imaging

For live imaging, pupae were mounted in halocarbon oil (series 700; Halocarbon Products) on a coverslip (Sigma) and imaged with a laser 510 scanning confocal microscope (ZEISS) at 25°C with a 40 × objective, using LSM Browser software (ZEISS). Z-stacks had a 3 *μ*m step size. Pupal collection and staging were done as described by [27].

#### Quantification

Landmark bristles were used to quantify cellular processes in the distal region as described by [15]. The changes in the apical cell area were measured using the subroutine “analyze particles” of the software ImageJ (NIH, http://rsb.info.nih.gov/ij). Apical cell boundaries were manually traced and the program calculated individual cell area. In addition, to compare the rate of expansion during rotation, we used as initial and final time points, 23 and 36 hours after pupariation (AP) respectively (unless otherwise specified).

## Supporting information

S1 Text Supporting information. (PDF)

S1 Video Frame-by-frame capture of the simulated SC rotation of Fig 2C. (AVI)

S2 Video Frame-by-frame capture of SC rotation of three flies with the same types as in Fig 4. (AVI)

S3 Video Frame-by-frame capture of simulated SC rotation of Fig 8B left. (AVI)

S4 Video Frame-by-frame capture of simulated SC rotation of Fig 8B right. (AVI)
