# Supplementary material for: Rotation of sex combs in Drosophila melanogaster requires precise and coordinated spatio-temporal dynamics from forces generated by epithelial cells
Source: PLoS Comput Biol. 2018 Oct 10;14(10):e1006455. doi: 10.1371/journal.pcbi.1006455 (PMC6179189; doi:10.1371/journal.pcbi.1006455)
Supplement: S1 Text — (PDF) [file pcbi.1006455.s001.pdf]

Supporting information for: Rotation of sex combs in *Drosophila melanogaster* requires precise and coordinated spatio-temporal dynamics from forces generated by epithelial cells

Ernest C. Y. Ho[1,a], Juan Nicolas Malagón[1,2,a], Abha Ahuja[3,4], Rama Singh[3], and Ellen Larsen[1]

[1]Department of Cell and Systems Biology, University of Toronto, Toronto, Ontario, Canada
[2]Boyer Center for Molecular Medicine, Yale University, New Haven CT, USA
[3]Department of Biology, McMaster University, Hamilton, Ontario, Canada
[4]College of Natural Sciences, Minerva Schools at KGI, San Francisco, California, USA
[a]Shared first authors

September 8, 2018

Number of Pages: 18 (including this title page)

Data analyses of experimental results of Figure 4

There are 5 experimental data points for each of ♀wt, ♂wt and ♂bab^{PR72} (Table A). Each data point consists of 4 coordinates: initial area of EP1, initial area of EP2, final area of EP1 and final area of EP2. We apply a combination of Monte Carlo simulations and bootstrapping to construct artificial data points based on these 5 actual experimental data points for each genotype for statistical analyses. First, we perform statistical bootstrapping: we construct 5000 extra sets of data points based on the 5 experimental data points. Each set of these data points consists of the same number of data points (5) as the original. Each data point of a set comes from random drawing of the 5 data points of the original experimental data set *with replacement*. Next, we perform Monte Carlo simulations on each of the 5000 bootstrapped data sets for each of ♀wt, ♂wt and ♂bab^{PR72}: for each of these bootstrapped data sets, we generate 1 million artificial data points from a multivariate Gaussian distribution with means and covariances identical to the means and covariances of that data set. Therefore, there are 4 sample mean values (each for initial EP1, initial EP2, final EP1, final EP2) and $4 \times 4 = 16$ covariance values arranged in a positive semi-definite symmetric matrix required for the generation of these 1 million artificial data points. Finally, we derive quantities and perform confidence interval estimates based on the 5000×3 realizations with a total of 15000 million artificial data points from the bootstrapped data sets of all three genotypes (both detailed below).

For each bootstrapped data set with the 1 million Monte Carlo simulated data points, we define the inhomogeneity coefficient $C_{inhomogeneity}$ as,

$$C_{inhomogeneity} \equiv \frac{Final\ area\ of\ EP2/Initial\ area\ of\ EP2}{Final\ area\ of\ EP1/Initial\ area\ of\ EP1}. \quad (1)$$

$C_{inhomogeneity}$ is a vector of 1 million elements. In equation 1, $C_{inhomogeneity}$ is formed by element-wise division of two vectors, $\frac{EP2_{Final}}{EP2_{Initial}}$ (numerator) and $\frac{EP1_{Final}}{EP1_{Initial}}$ (denominator), which are themselves formed by element-wise division of the respective individual data points within

the same bootstrapped set. For example, the first element of $\frac{EP2_{Final}}{EP2_{Initial}}$ is obtained by dividing the value of the final area of EP2 of the first data point with the initial area of EP2 of the first data point. Therefore, an element of $C_{inhomogeneity} > 1$ means that, for that particular simulated data point, EP2 expands more (or contracts less) as a proportion of its initial size than EP1, and *vice versa* for an element of $C_{inhomogeneity} < 1$. Thus, $C_{inhomogeneity}$ captures the inhomogeneity of distal cell expansion during SC rotation.

To explore the effects of being ♀wt, ♂wt or ♂bab^{PR72} has on the inhomogeneity of expansion of distal cells, we define $\Delta C_{inhomogeneity}$ as the difference of $C_{inhomogeneity}$ between any of the two of ♂wt, ♀wt and ♂bab^{PR72} (therefore, two bootstrapped data sets, each of 1 million artificial data points, are required for arguments of $\Delta C_{inhomogeneity}$):

$$\Delta C_{inhomogeneity}(s1, s2) \equiv C_{inhomogeneity}(s1) - C_{inhomogeneity}(s2). \tag{2}$$

Thus, $\Delta C_{inhomogeneity}(s1, s2)$ (a vector of 1 million elements) captures how much such an asymmetry of distal cell expansion varies between $s1$ and $s2$ where $s1$ and $s2$ can be any of ♀wt, ♂wt and ♂bab^{PR72}. In obtaining $\Delta C_{inhomogeneity}(s1, s2)$, we randomly shuffle the elements of $C_{inhomogeneity}(s2)$ before the vector subtraction to avoid potential artifacts resulting from the seeds of the pseudo random number generator. Figure A shows how the values of $C_{inhomogeneity}$, $\Delta C_{inhomogeneity}(s1, s2)$ and other related quantities based on three example bootstrapped data sets can be visualized as distributions. For statistical analyses, there is a total of 5000$\times$ 3 such $C_{inhomogeneity}$ distributions. We have also used 5000$\times$ 3 such $\Delta C_{inhomogeneity}(s1, s2)$ distributions (5000 for each pair of $(s1, s2), s1 \neq s2$), out of a total of 3$\times$ 5000 $\times$ 5000 possible combinations, not counting interchange of indices.

In the main text, we used derived quantities such as $Pr(\frac{EP2_{Initial}}{EP1_{Initial}} < 1)$ or $Pr(C_{inhomogeneity} > 1)$. These probability values for each realization of bootstrapped data sets are defined as the proportion of area of the relevant distribution that satisfies the said criterion. These probability values are themselves random variables with a probability distribution that can be visualized by calculating every realization of the probability value from every bootstrapped data set.

Thus, confidence intervals of these probability values and other derived quantities can be estimated in such a manner (Table B).

Data analyses of ABASCT SD statistics of simulated intact SC without temporal dynamics

We first perform ANOVA on the ABASCT SD data of simulated intact SC without temporal dynamics (i.e. delay=0 mcs) to test the equality of the means of ABASCT SD values between SC lengths (5, 7, 9 or 11-tooth SCs). The resulting F-test statistic has a value of 120 with Pr(>F)$< 2 \times 10^{-16}$, which shows at least one of the means is significantly different from the other three. Post-hoc Tukey's test is then used to pairwise compare the ABASCT SD data of each group (SC lengths). The results are shown in Table C and Figure E.

Calculation of p-value in Table D

In Table D we estimated the p-value between the breaking statistics of two sets of SC simulations. Given that $0 \le A \le B \le 48$, the p-values quoted in Table D are related to the probability that any two sets of simulations with intact ratio of at most $\frac{A}{48}$ and intact ratio of at least $\frac{B}{48}$ share a common probability distribution. These values are calculated as follows.

Assuming that each simulation is independent with the rotated SC having a probability P of being intact, in a set of 48 simulations the probability of getting A intact SCs is thus,

$$f(A, 48, P) = \binom{48}{A} \times P^A \times (1 - P)^{48-A} \quad (3)$$

where

$$\binom{48}{A} \equiv \frac{48!}{A! \times (48 - A)!}. \quad (4)$$

Therefore, the probability that a set of simulations has at most A intact SCs is simply,

$$F_1(A, 48, P) = \sum_{i=0}^{A} f(i, 48, P). \tag{5}$$

Similarly, the probability for a set of simulations to get at least B intact SCs is,

$$F_2(B, 48, P) = \sum_{i=B}^{48} f(i, 48, P). \tag{6}$$

Multiplying equations 5 and 6 gives the probability that both events happen together,

$$G(A, B, 48, P) = F_1(A, 48, P) \times F_2(B, 48, P). \tag{7}$$

To derive the p-value, one needs the maximum of $G(A, B, 48, P)$ along the P-axis ("worst case scenario"). One method to find such values is to differentiate $G(A, B, 48, P)$ with respect to P and set the expression to zero,

$$\frac{dG(A, B, 48, P)}{dP} = 0 \tag{8}$$

and look for the values of P that solve equation 8 and satisfy the second derivative condition $\frac{d^2 G(A,B,48,P)}{dP^2} < 0$. Once we get these values we can determine whether any of those values of P or the "end-point" values (i.e. $P = 0$ or 1) yields the maximum of equation 7 for $0 \leq P \leq 1$. In our case, we use an alternative numerical approach of scanning the numerical maximal value of G by increasing $0 \leq P \leq 1$ step-wise. The p-value reported in each row of Table D is two times this numerical maximum.

Figure A: Example distributions of Monte Carlo simulated quantities.

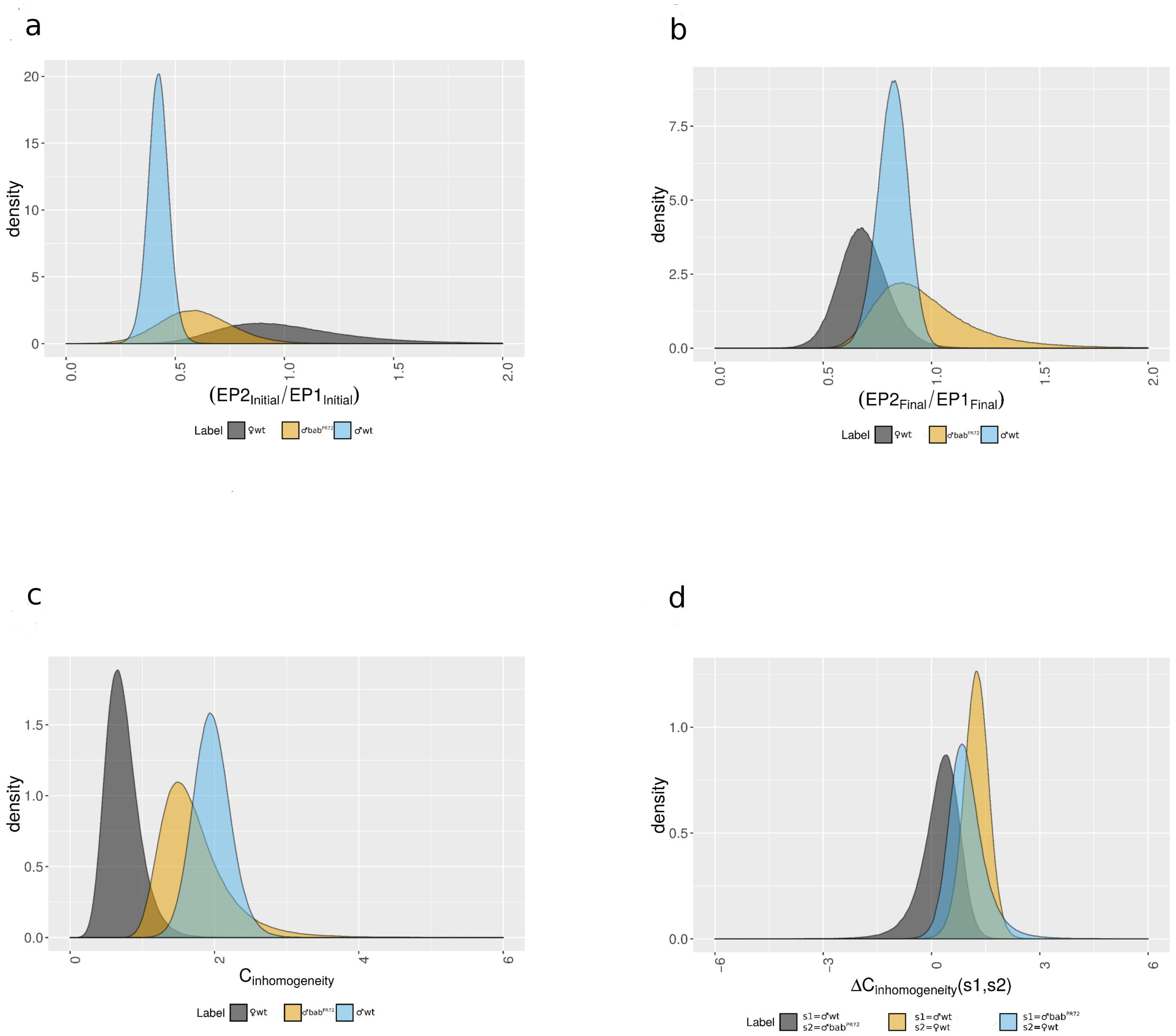

a Example distributions of Monte Carlo simulated $\frac{Initial\ area\ of\ EP2}{Initial\ area\ of\ EP1}$ for ♀wt, ♂wt and ♂ bab^{PR72}.
b Example distributions of Monte Carlo simulated $\frac{Final\ area\ of\ EP2}{Final\ area\ of\ EP1}$ for ♀wt, ♂wt and ♂ bab^{PR72}.
c Example distributions of Monte Carlo simulated $C_{inhomogeneity}$ for ♀wt , ♂wt and ♂ bab^{PR72}.
d Example distributions of Monte Carlo simulated $\Delta C_{inhomogeneity}(s1, s2)$.

Figure B: Histograms of angle of rotation α of all simulated intact SCs without temporal dynamics. Each histogram shows counts of a single SC length (5, 7, 9 or 11-tooth SC).

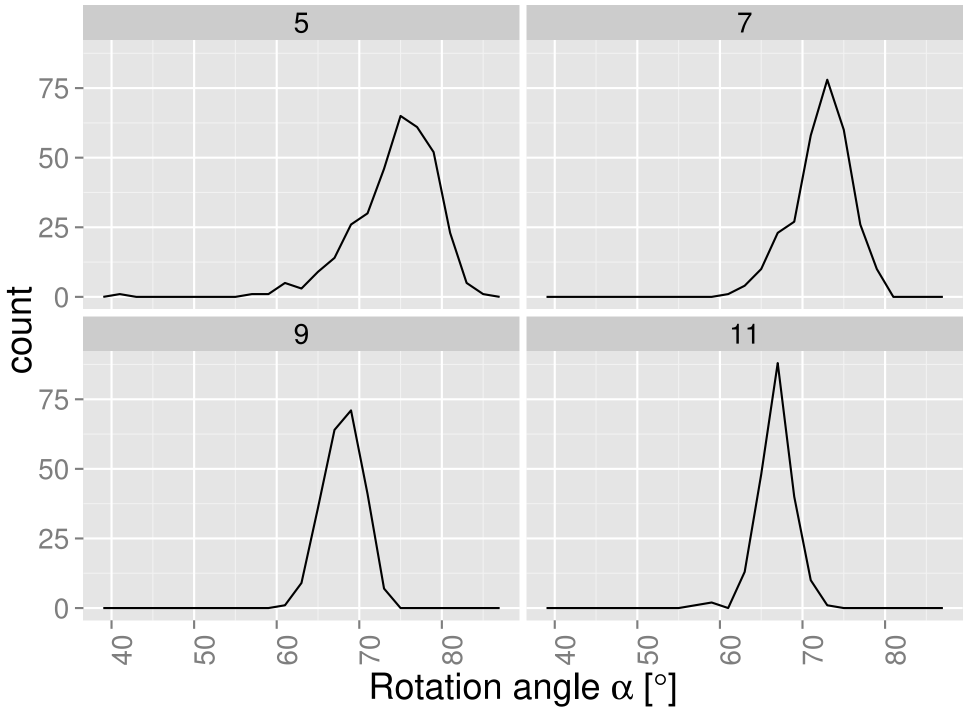

Figure C: Histograms of ABASCT SD of all simulated intact SCs without temporal dynamics. Each histogram shows counts of a single SC length (5, 7, 9 or 11-tooth SC).

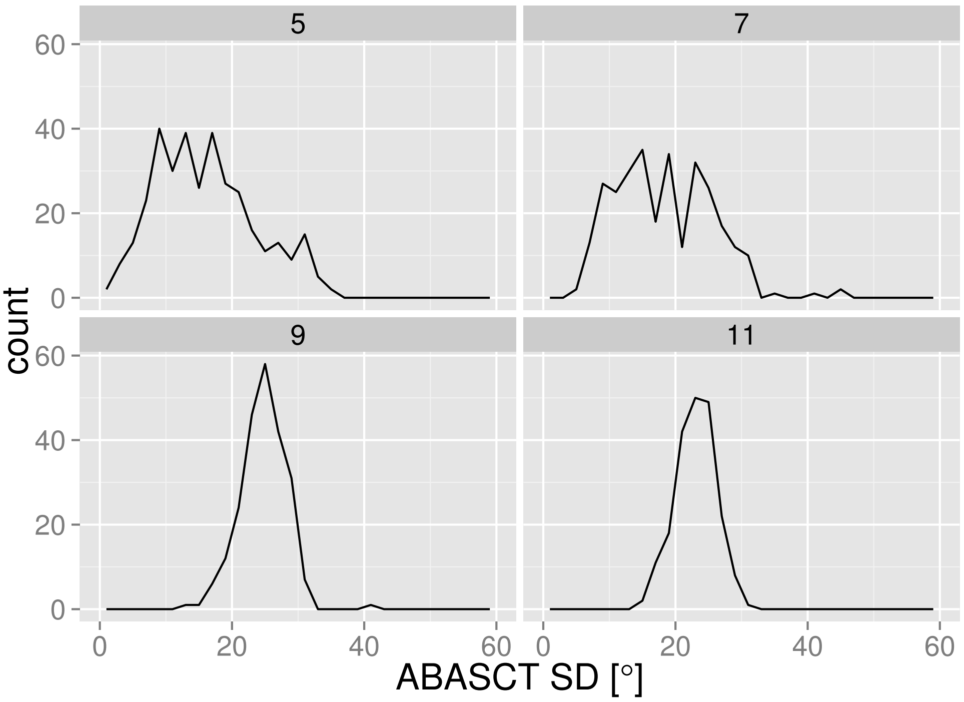

Figure D: Graph of aggregate ABASCT SD statistics of simulated and intact rotated SCs without temporal dynamics, grouped by adhesion parameters. This graph is based on the same data as Fig 7B, except here the horizontal centre line of each box is the mean. Height of each box from the horizontal centre line represents $\pm$ 1SEM (standard error of the mean). The upper (lower) extent of each top (bottom) whisker represents the maximum (minimum) ABASCT SD value of that category.

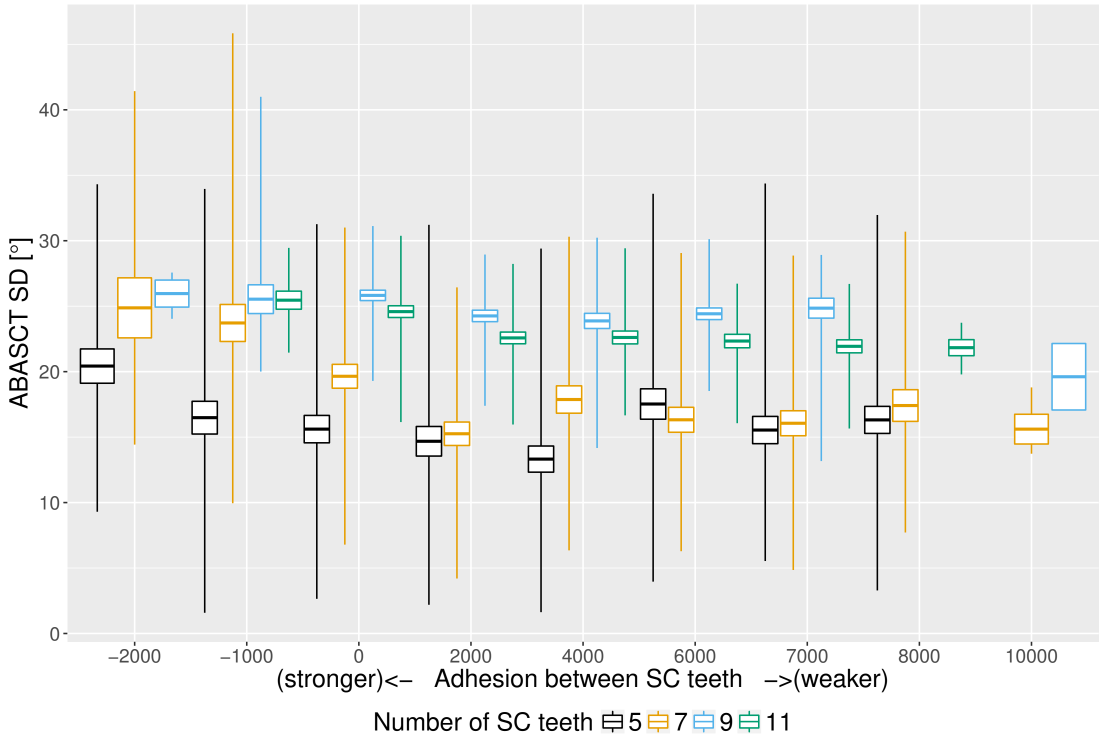

Figure E: Linear model fit of treatment (number of SC teeth) effects on ABASCT SDs of simulated intact SCs without temporal dynamics.

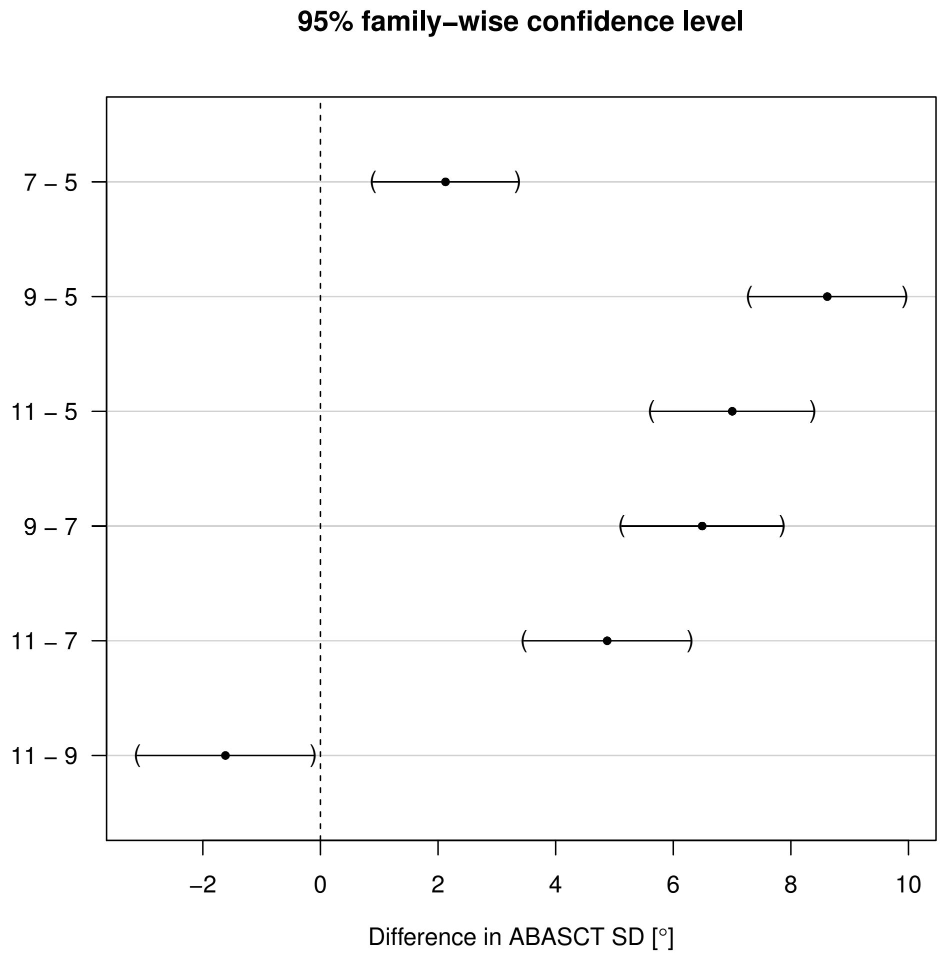

Figure F: Box plot of ABASCT SD of intact SC simulations without temporal dynamics, but with regular 5-tooth simulations replaced by the “slowed down” version, grouped by adhesion between SC teeth.

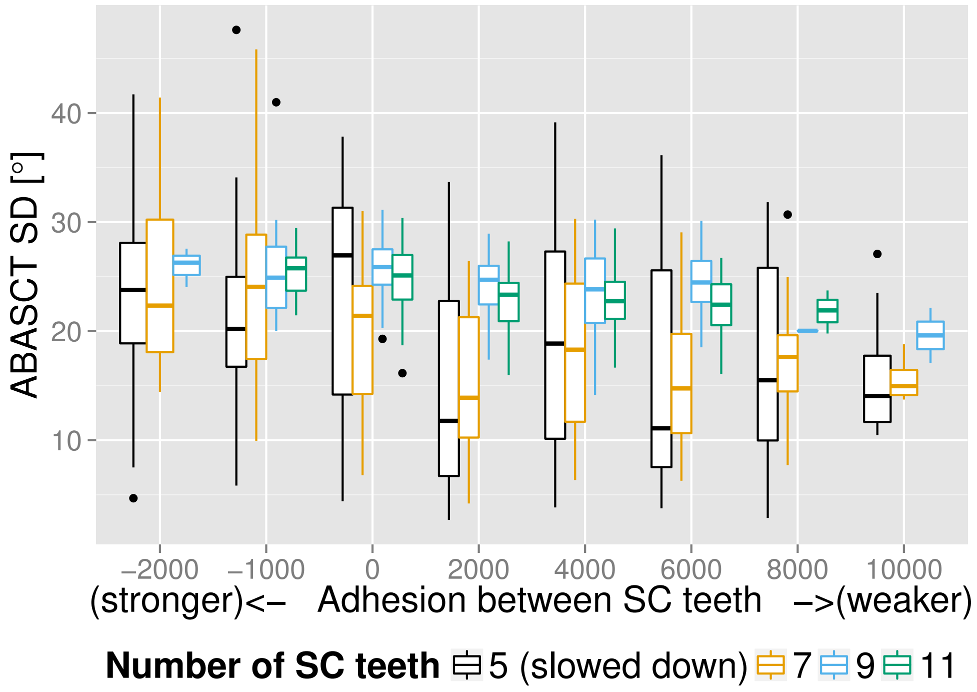

Figure G: Histograms of angle of rotation α of all simulated intact SCs grouped by magnitude of delayed expansion of EP1 cells (delay=0 means absence of temporal dynamics). Each histogram shows counts of a single SC length (5, 7, 9 or 11-tooth SC).

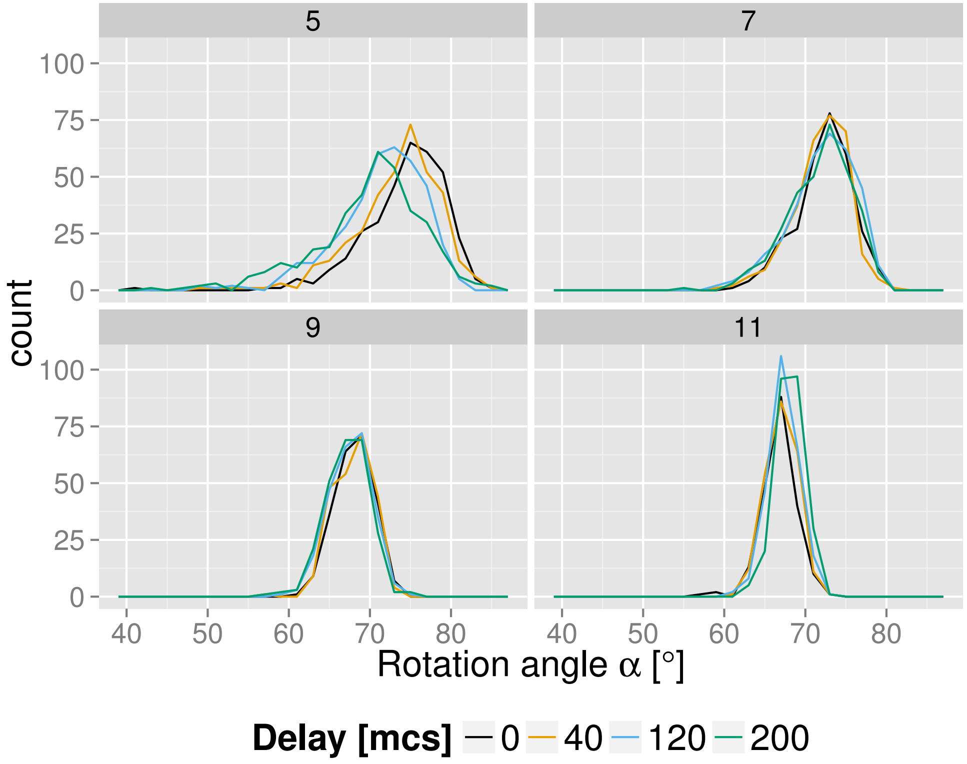

Figure H: Histograms of ABASCT SD of all simulated intact SCs grouped by magnitude of delayed expansion of EP1 cells (delay=0 means absence of temporal dynamics). Each histogram shows counts of a single SC length (5, 7, 9 or 11-tooth SC).

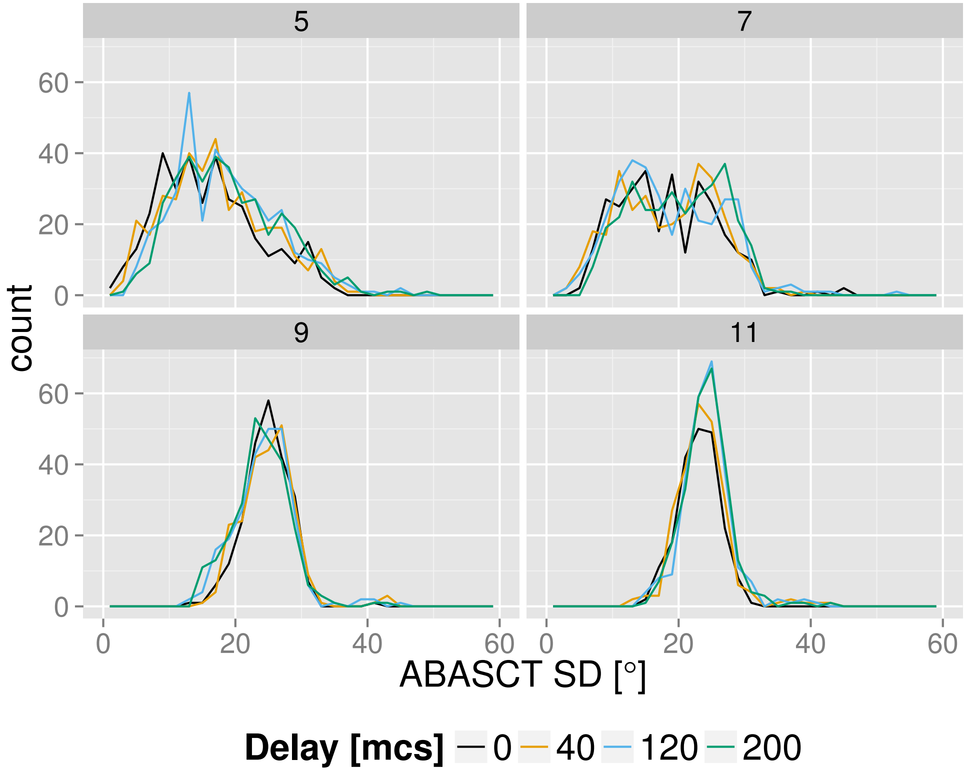

Figure I: An example 9-tooth SC simulation which uses 4 times as many pixels as the original ones.

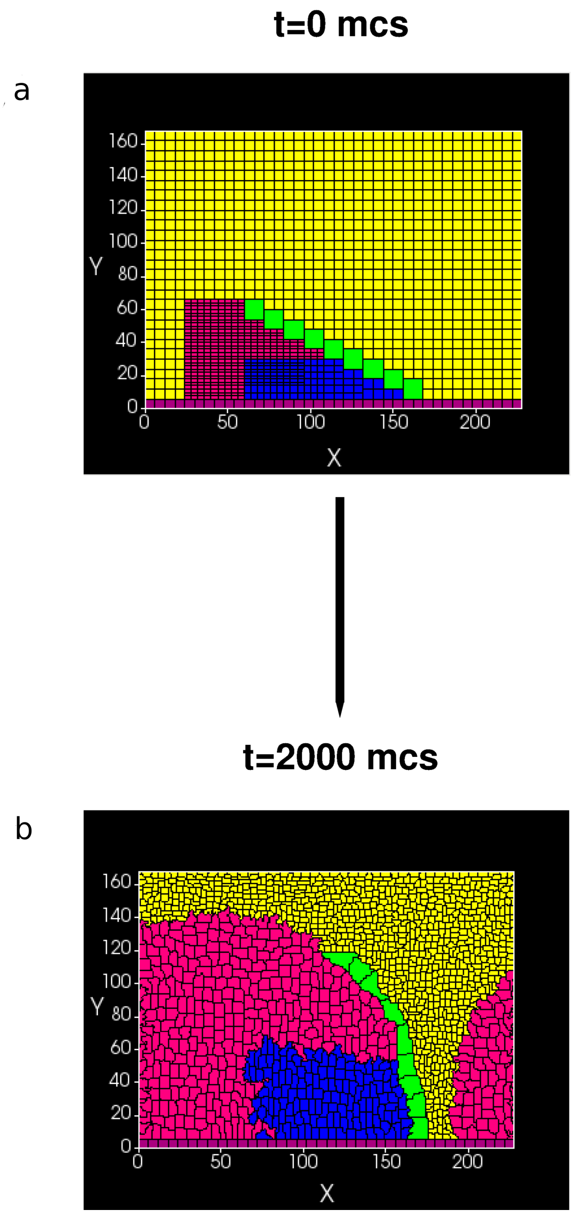

a Start of simulation (t=0 mcs).
b End of simulation (t= 2000 mcs).

Figure J: Confocal image of an example fly leg showing the results of individual cell tracking according to two cell labelling definitions.

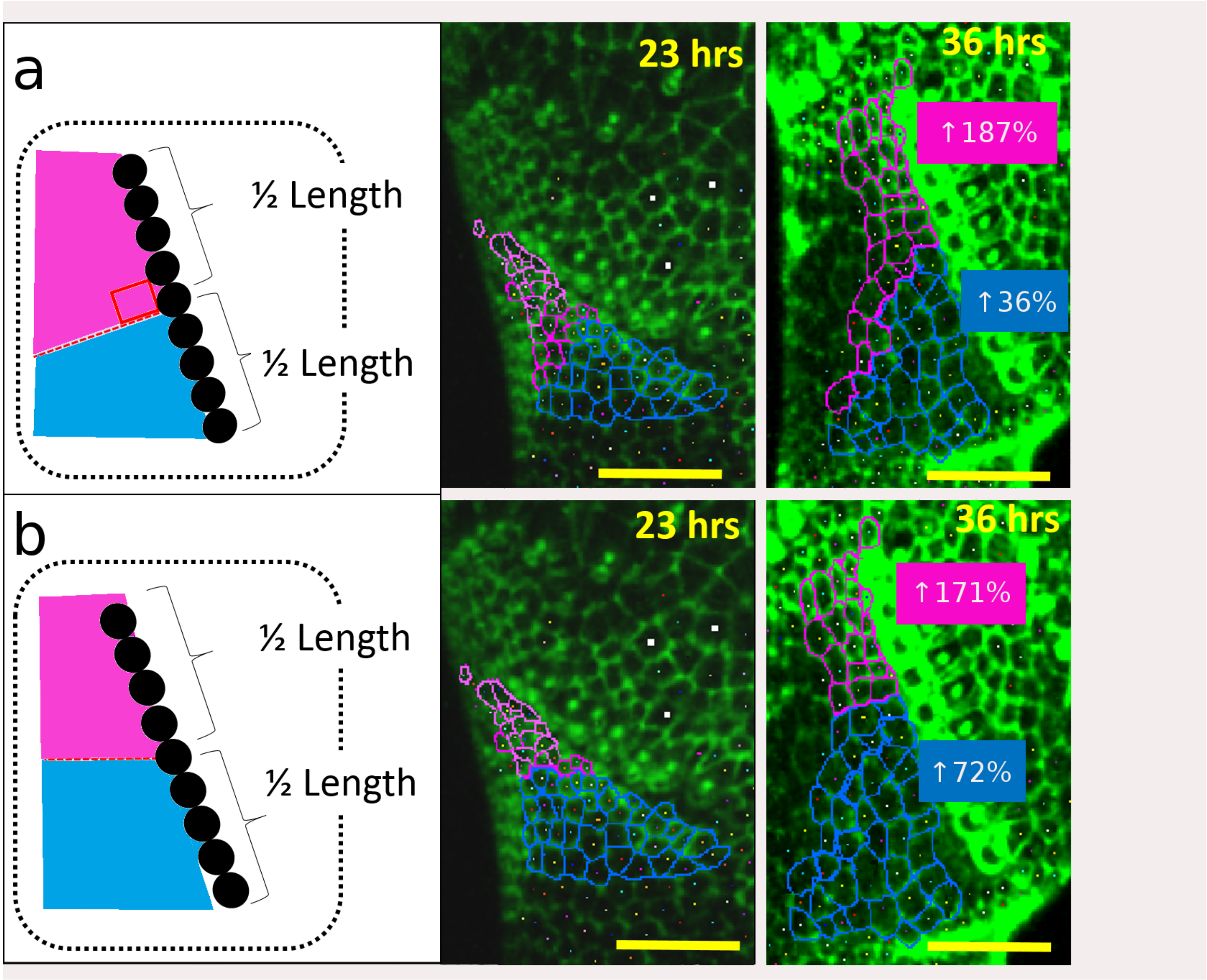

a The demarcation line is drawn perpendicular to the SC at the halfway length mark of the SC at 23 hours AP.
b The demarcation line is drawn horizontally at the halfway length mark of the SC at 23 hours AP.
Regardless of definitions, the EP2 cells (magenta) on average expand much more than EP1 cells (blue). For example, in **a**, the increase in area of EP2 cells is 187% vs. EP1 cells at 36%. Each scale bar of **a** and **b**: 20 μm.

Tables

Fly type	Pupa Number	Initial EP1 [μm^2]	Initial EP2 [μm^2]	Final EP1 [μm^2]	Final EP2 [μm^2]
♀wt	1	6.4	6.3	6.9	5.0
♀wt	2	5.8	5.9	6.7	5.3
♀wt	3	6.6	3.7	5.7	3.8
♀wt	4	3.5	5.7	4.2	3.1
♀wt	5	5.9	5.8	7.1	3.6
♂ bab^{PR72}	1	4.1	2.3	6.6	6.8
♂ bab^{PR72}	2	5.3	2.1	11.4	7.5
♂ bab^{PR72}	3	4.7	3.5	9.3	8.6
♂ bab^{PR72}	4	5.1	4.0	7.2	8.1
♂ bab^{PR72}	5	5.6	2.6	6.7	7.3
♂wt	1	3.6	1.6	9.1	8.2
♂wt	2	2.9	1.4	9.0	6.3
♂wt	3	3.3	1.1	9.9	7.7
♂wt	4	4.8	2.0	9.8	8.1
♂wt	5	4.2	1.9	10.5	9.3

Table A: Raw experimental data of area measurement in Figure 4.

Quantity (↓); Quantile (→)	0%	25%	50%	75%	100%
$Pr(\frac{EP2_{Initial}}{EP1_{Initial}} < 1)$ ♀wt	0.03	0.36	0.54	0.69	1.00
$Pr(\frac{EP2_{Initial}}{EP1_{Initial}} < 1)$ ♂bab^{PR72}	0.95	0.98	0.99	1.00	1.00
$Pr(\frac{EP2_{Initial}}{EP1_{Initial}} < 1)$ ♂wt	1.00	1.00	1.00	1.00	1.00
median $\frac{EP2_{Initial}}{EP1_{Initial}}$ ♀wt	0.56	0.89	0.97	1.07	1.45
median $\frac{EP2_{Initial}}{EP1_{Initial}}$ ♂bab^{PR72}	0.40	0.54	0.59	0.63	0.78
median $\frac{EP2_{Initial}}{EP1_{Initial}}$ ♂wt	0.33	0.41	0.43	0.44	0.48
$Pr(C_{inhomogeneity} > 1)$ ♀wt	0.00	0.01	0.14	0.24	1.00
$Pr(C_{inhomogeneity} > 1)$ ♂bab^{PR72}	0.90	0.98	1.00	1.00	1.00
$Pr(C_{inhomogeneity} > 1)$ ♂wt	0.97	0.99	1.00	1.00	1.00
median $C_{inhomogeneity}$ ♀wt	0.47	0.64	0.70	0.76	1.19
median $C_{inhomogeneity}$ ♂bab^{PR72}	1.27	1.53	1.65	1.76	2.35
median $C_{inhomogeneity}$ ♂wt	1.45	1.87	1.94	2.01	2.33
$Pr(\Delta C_{inhomogeneity}($♂$wt,$ ♂$bab^{PR72}) > 0)$	0.03	0.59	0.70	0.81	1.00
$Pr(\Delta C_{inhomogeneity}($♂$wt,$ ♀$wt) > 0)$	0.88	0.99	1.00	1.00	1.00
$Pr(\Delta C_{inhomogeneity}($♂$bab^{PR72},$ ♀$wt) > 0)$	0.75	0.96	0.99	1.00	1.00
median $\Delta C_{inhomogeneity}($♂$wt,$ ♂$bab^{PR72})$	-0.48	0.13	0.27	0.41	0.87
median $\Delta C_{inhomogeneity}($♂$wt,$ ♀$wt)$	0.48	1.11	1.23	1.34	1.70
median $\Delta C_{inhomogeneity}($♂$bab^{PR72},$ ♀$wt)$	0.26	0.82	0.95	1.08	1.74

Table B: Monte Carlo simulated quantities on bootstrapped data sets.

A	B	Estimate [°]	Error [°]	t value	Pr(>|t|)
7	5	2.13	0.48	4.43	<0.001***
9	5	8.62	0.52	16.69	<0.001***
11	5	7.00	0.54	13.07	<0.001***
9	7	6.49	0.53	12.2	<0.001***
11	7	4.88	0.55	8.85	<0.001***
11	9	-1.62	0.59	-2.77	0.029*

Table C: Results of Tukey post-hoc hypothesis testing on the equality of treatments (number of SC teeth) on ABASCT SDs of simulated intact SCs without temporal dynamics. The (null) hypothesis $H_0(A, B)$ tested in each row is that there is "no treatment effect on ABASCT SDs between A-tooth SCs and B-tooth SCs (informally, A-B=0)".

A	Delay parameter used in set with A intact SCs [mcs]	B	Delay parameter used in set with B intact SCs [mcs]	Length	$J(SCT, SCT)$	p-value
43	0	48	120	11-tooth	0	≤0.0082**
41	0	47	120	11-tooth	2000	≤0.0082**
38	0	42	120	11-tooth	4000	≤0.077
38	0	43	200	11-tooth	4000	≤0.046*
32	0	37	120	11-tooth	6000	≤0.068
32	0	41	200	11-tooth	6000	≤0.0076**
6	0	17	120	11-tooth	8000	≤0.0019**

Table D: Results of hypothesis testing on the equality of SC breaking probabilities between two sets of simulations. The (null) hypothesis $H_0(A, B)$ tested in each row is "a simulation set that has at most A intact SCs and another simulation set that has at least B intact SCs share a common probability distribution of SC breakage".
